# Polyploid superficial uroepithelial bladder barrier cells express features of cellular senescence across the lifespan and are insensitive to senolytics

**DOI:** 10.1111/acel.14399

**Published:** 2024-12-07

**Authors:** Iman M. Al‐Naggar, Maria Antony, Dylan Baker, Lichao Wang, Lucas Da Cunha Godoy, Chia‐Ling Kuo, Matthew O. Fraser, Phillip P. Smith, Ming Xu, George A. Kuchel

**Affiliations:** ^1^ Center on Aging University of Connecticut Farmington Connecticut USA; ^2^ Department of Cell Biology University of Connecticut Health Farmington Connecticut USA; ^3^ Department of Surgery University of Connecticut Health Farmington Connecticut USA; ^4^ The University of Connecticut School of Medicine Farmington Connecticut USA; ^5^ Department of Genetics & Genome Sciences University of Connecticut Health Farmington Connecticut USA; ^6^ The Jackson Laboratory for Genomic Medicine Farmington Connecticut USA; ^7^ The Cato T. Laurencin Institute for Regenerative Engineering Farmington Connecticut USA; ^8^ Department of Research & Development Durham Veterans Affairs Medical Centers Durham North Carolina USA; ^9^ Connecticut Institute for Brain and Cognitive Science University of Connecticut Storrs Connecticut USA

**Keywords:** aging, beneficial cellular senescence, bladder, blood‐urine barrier, D + Q, lower urinary tract dysfunction, LUTS, senolytics, tetraploidy‐induced senescence, umbrella cells

## Abstract

Lower urinary tract dysfunction (LUTD) increases with aging. Ensuing symptoms including incontinence greatly impact quality of life, isolation, depression, and nursing home admission. The aging bladder is hypothesized to be central to this decline, however, it remains difficult to pinpoint a singular strong driver of aging‐related bladder dysfunction. Many molecular and cellular changes occur with aging, contributing to decreased resilience to internal and external stressors, affecting urinary control and exacerbating LUTD. In this study, we examined whether cellular senescence, a cell fate involved in the etiology of most aging diseases, contributes to LUTD. We found that umbrella cells (UCs), luminal barrier uroepithelial cells in the bladder, show senescence features over the mouse lifespan. These polyploid UCs exhibit high cyclin D1 staining, previously reported to mediate tetraploidy‐induced senescence in vitro. These senescent UCs were not eliminated by the senolytic combination of Dasatinib and Quercetin. We also tested the effect of a high‐fat diet (HFD) and senescent cell transplantation on bladder function and showed that both models induce cystometric changes similar to natural aging in mice, with no effect of senolytics on HFD‐induced changes. These findings illustrate the heterogeneity of cellular senescence in varied tissues, while also providing potential insights into the origin of urothelial cancer. We conclude that senescence of bladder uroepithelial cells plays a role in normal physiology, namely in their role as barrier cells, helping promote uroepithelial integrity and impermeability and maintaining the urine‐blood barrier.

## Graphical abstract

This study characterized umbrella cells, polyploid bladder uroepithelial barrier cells, as an example of potentially beneficial senescent cell present under normal physiology throughout the lifespan in mouse bladders. Bladder umbrella cells exhibit markers of tetraploidy‐induced senescence. They are resistant to depletion by the senolytic combination of Dasatinib and Quercetin. Both high‐fat diet and senescent cell transplantation were used as models of accelerated aging and caused bladder functional changes similar to those observed with natural aging in mice.

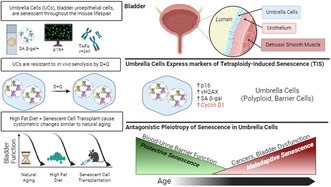

**Abbreviations**
BSA: bovine serum albumin
CMG: pressure/flow cystometry
D+Q: Dasatinib and Quercetin
DSM: detrusor smooth muscle
FDR: False Discovery Rate
γH2AX: phosphorylated histone 2AX
HFD: high fat diet
IP: intraperitoneally
LUTD: lower urinary tract dysfunction
LUTS: lower urinary tract symptoms
mAB: monoclonal antibody
NCD: normal chow diet
NGS: normal goat serum
OCT: Optimal Cutting Temperature
PBS: phosphate buffered saline
Pmax: maximum void‐associated pressure
RT‐qPCR: reverse transcription quantitative polymerase chain reaction
RNA FISH: RNA Fluorescence in situ hybridization (RNAscope)
SA β‐gal: senescence‐associated β‐galactosidase assay
SASP: senescence‐associated secretory phenotype
SnCs: senescent cells
scRNA‐seq: single cell RNA sequencing
TAFs: telomere associated foci
UCs: umbrella cells
UMAP: Uniform Manifold Approximation and Projection
VSA: voiding spot assays

## 1 INTRODUCTION

Lower urinary tract symptoms (LUTS), including urinary incontinence, frequency, urgency, and nocturia, are highly prevalent in older adults (Nordling, 2002). They affect both sexes and greatly contribute to loss‐of independence, increased risk of falls and fractures, social isolation, and depression (Vaughan et al., 2018). In addition, LUTS place a huge burden on our economy (Coyne et al., 2014), and palliative measures in the form of disposable adult incontinence products contribute to environmental pollution. Current therapies have focused on targeting defined signaling pathways in detrusor smooth muscle (DSM) function, with the assumption that, like skeletal muscle, it loses functionality with aging. Antimuscarinics and the β3‐adrenoceptor agonist are suboptimal, and ineffective in dysfunction of aging such as Detrusor Hyperactivity with Impaired Contractility (DHIC) (Gibson et al., 2021). This inefficacy, concomitant with high cost and undesirable side effects, makes it so that these drugs are usually quickly abandoned by patients (Benner et al., 2010). In addition, anticholinergic drug use has been linked with concerns regarding cognitive compromise (Gray et al., 2015).

Bladder function is unique in that it is a rare example of a brainstem/autonomic control system being subject to an overarching cortical/voluntary control. Mounting evidence supports a role for the aging brain in bladder dysfunction (Zhao et al., 2022), with decreased bladder volume sensations (Pfisterer et al., 2006). In addition, aging‐related pathologies such as atherosclerosis, leading to ischemia in the bladder, inflammation, and oxidative stress, can result in bladder denervation, cellular loss, tissue damage, and excessive deposition of extracellular matrix leading to fibrosis and loss of elasticity (Kullmann et al., 2015). Mitochondrial dysfunction also likely plays a role (de Rijk et al., 2022). Aging‐related bladder dysfunction is a multifactorial, complex issue, and is unlikely to be eliminated by treatments targeting just one of those aspects. Interest has thus shifted to a more systemic, rather than organ‐based, approach to the study, diagnosis, and treatment of bladder dysfunction. Consequently, aging‐related bladder dysfunction may be a perfect candidate for testing and applying the Geroscience Hypothesis with possible benefit from treatments such as senolytics, rapamycin, and metformin that can simultaneously target and improve several underlying hallmarks of biological aging (Kennedy et al., 2014; Tchkonia et al., 2013).

Animal studies investigating biologic aging pathways demonstrate the importance of senescent cells in age‐associated physiologic dysfunction (Baker et al., 2016; Suryadevara et al., 2024; Wang et al., 2021). Senescent cells (SnCs) are cells that have permanently exited the cell cycle, in response to stress and accumulation of damage (Gasek et al., 2021; Suryadevara et al., 2024). This process is believed to have evolved as a protective mechanism against malignant transformation (Campisi, 2013). SnCs are resistant to apoptosis and produce many inflammatory molecules, including chemokines and cytokines, which are important for their clearance by the immune system (Kirkland & Tchkonia, 2017). However, with aging, the immune system fails to properly respond to these signals, causing SnCs to accumulate and continue to secrete debilitating factors, resulting in propagation of senescence, systemic inflammation, tissue damage, ultimately leading to diseases such as cancer, organ, and stem cell dysfunction.

Agents aimed at removing (senolytics) or modulating (senomorphics) SnCs, have been shown to prevent or delay the onset and progression of declines involving frailty, mobility, metabolic dysfunction, cardiac performance, immune defense, bone loss, and cognition in mouse models (Al‐Naggar et al., 2020). Translational approaches using senolytics are increasingly common, yet the current study represents the first examination of using these novel therapeutics as a means of targeting voiding disorders seen with aging.

In the current study, we set out to investigate and characterize for the first time SnCs in an aging mouse model and test the ability of the senolytic combination of Dasatinib (D) + Quercetin (Q), D + Q, to prevent or reverse SnC accumulation and related bladder functional declines seen in old mice. We hypothesized that SnCs, either acting systemically or within the bladder, contribute to aging‐associated declines in lower urinary tract function, thus predisposing to dysfunction. We also proposed that removing SnCs could slow or reverse such declines.

We now report that surface luminal uroepithelial umbrella cells appear to be the only cell population demonstrating multiple senescence features in the mouse bladder. However, despite increases in some of these parameters with maturation and/or aging, most were observed throughout the lifespan. Based on published ex vivo studies, polyploid cells such as these umbrella cells may undergo premature senescence via oncogene activation, as previously described for “tetraploidy‐induced senescence” (Panopoulos et al., 2014). Furthermore, these cells were not cleared using a standard and widely used senolytic regimen that has successfully been used to clear SnCs in several organ systems in mice both by our labs and others. With all these considerations in mind, we propose that the senescence of these cells throughout the lifespan and their apparent resistance to standard senolytic regimen may in fact represent an example of “beneficial senescence”, ensuring the integrity and maintenance of a key blood‐urine barrier by these UCs (de Magalhaes, 2024). Finally, we hypothesize that these polyploid senescent UCs, by escaping senescent cell arrest and becoming aneuploid, may give rise to bladder cancers of urothelial origin, potentially illustrating a novel example of antagonistic pleiotropy of cellular senescence.

## 2 RESULTS

### 2.1 Identifying senescent cells in the mouse bladder

**FIGURE 1:**
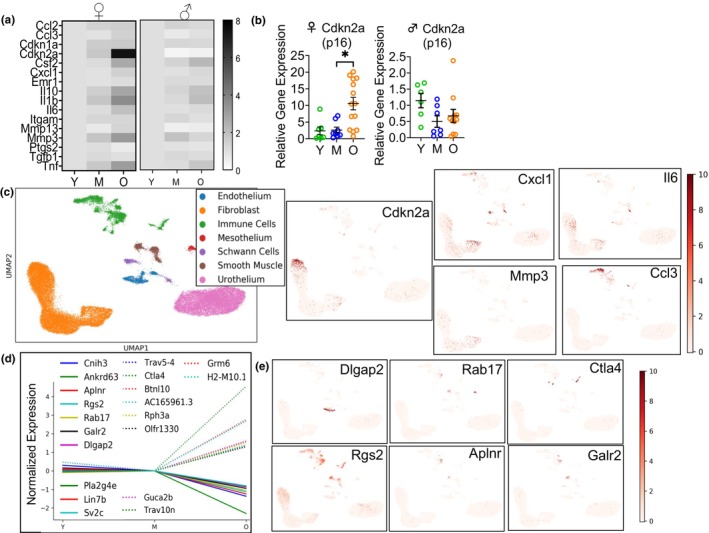
Transcriptomic Characterization of Senescence and Aging in the Mouse Bladder. (a) Heat map of RT‐qPCR for senescent cell markers, SASP factors and immune cells in young (Y), middle aged (M) and old (O) female (left) and male (right) mouse bladders. *N* = 6–14 per age group for each sex. Relative gene expression calculated using geometric means shown. (b) RT‐qPCR data for p16 in female and male bladders over the lifespan. Kruskal–Wallis test with Dunn's correction for multiple comparisons. *p < 0.05 compared to Middle aged group. (c) Single cell sequencing data using pooled mouse bladders from different age groups to identify cells expressing senescent cell markers, SASP factors and genes whose expression changed significantly from middle to old age in our whole bladder RNA sequencing data. Top left, Cell type annotation of previous scRNA‐seq data performed on mouse bladders (GSE180128); Center right and Bottom, Expression of genes that were found to be differentially expressed in whole bladder RT‐qPCR data. (d) Whole bladder RNA sequencing data performed on young (Y, 2 months), middle‐aged (M, 10 months), and old (O, 26 months) mouse bladders. Displayed genes are those significant genes (adj. *p*‐value <0.05) with the highest |fold change| between middle‐aged and old bladders which have minimal change (|fold change| <0.2) between young and middle‐aged bladders. Expression (TPM) normalized to the middle aged group (*n* = 4–6 per age group). (e) Expression of genes found to be differentially expressed (|fold change| >1.5, adj *p*‐value <0.05) between old and middle samples in bulk RNA sequencing plotted in previous scRNA‐seq data. Example genes chosen based on intersection of significant bulk RNA seq gene set with marker genes identified from previous scRNA‐seq (GSE180128).

**FIGURE 2:**
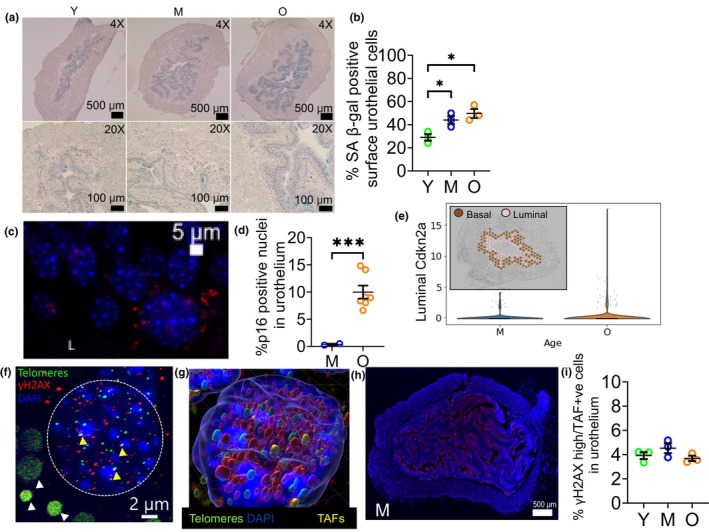
Identification of Senescent Cell Types in the Mouse Bladder. (a) Senescence Associated β‐galactosidase assay in female mouse bladder sections over the lifespan (Y = young (2–4 months), M = middle aged (10–12 months), O = old (20–26 months)). (b) Quantification of Senescence Associated β‐galactosidase assay in female mouse bladder sections over the lifespan. *N* = 3 mice/age group. Ordinary one‐way ANOVA, with Tukey test for multiple comparisons, *significant *p* < 0.05. (c) Representative image of P16 RNAscope in old mouse bladder surface urothelial cell. (d) Quantification of p16 RNAscope in middle aged and old mouse bladder urothelial nuclei. Unpaired *t* test with Welch's correction, two‐tailed, *significant *p* < 0.05, **p < 0.005, ***p < 0.0005. (e) Spatial transcriptomics data (Visium) (from GSE180128) with luminal urothelial cells isolated and expression of *Cdkn2a* in luminal urothelium compared between middle aged and old bladder section (*n* = 4, *p*‐value = 0.0349). (f) Telomere‐associated foci (TAFs) in a surface urothelial umbrella cell. Yellow arrowheads show colocalized telomere and γH2AX fluorescence, whereas white arrowheads show auto‐fluorescent lipofuscin droplets, the aging pigment, in cytoplasm of these same TAF positive cells. (g) IMARIS‐generated image identifying TAFs (yellow) based on overlap between surfaces created around telomere (green) and γH2AX (red) fluorescence. (h) Whole female mouse bladder section γH2AX staining (red), 20X stitched, showing high γH2AX expression in senescent surface luminal cells. (i) Quantification of TAF positive cells in urothelium in female mouse bladder sections over the lifespan *N* = 3 mice/age group. Ordinary one‐way ANOVA with Tukey's test for multiple comparisons, *significant *p* < 0.05.

We began our study of SnCs in the mouse bladder by performing transcriptomics studies to look for aging and SnC gene signatures over the mouse lifespan. We performed RT‐qPCR on mouse bladders from both female and male mice at 3 age groups: Young (Y, 2–6 months), Middle‐aged (M, 10–14 months) and Old (O, 18–26 months). Whereas we observed a significant age‐related increase in several transcripts often associated with senescence (e.g., *Cdkn2a*, *Ccl3*, *Cxcl1*, *Il‐6*, *Mmp3*, *Mmp13*, *Csf2*, *Il10*, and *TNF*) in female mice over the lifespan, these changes were not observed in male mice over the same ages tested (Figure 1a,b, and Figure S1). Several senescence markers and senescence‐associated secretory phenotype (SASP) factors are also markers of inflammation, so we sought to determine whether this increase in markers of senescence correlated with an increase in bladder immune cell population, often seen in the aging bladder (Ligon et al., 2020). There were no significant increases in mRNA detected for immune cell‐specific transcripts (e.g., *Emr1*, *Itgam*), suggesting the observed increase in inflammatory molecules is not due to increased immune cell populations. The surprising lack of transcriptomic senescence signature in the male mouse bladder with aging prompted us to pursue our studies in female mice. To identify other genes whose expression changes with aging in mouse bladders, we performed bulk RNA sequencing of female mouse bladders over the lifespan (Y, M, O). We observed a similar increase in senescence and inflammation markers from middle to old age as seen with RT‐qPCR (Figure S2). We additionally identified genes whose expression changed significantly (False Discovery Rate, FDR <0.05) between middle‐aged (mature) and old mouse bladders and displayed minimal change between young and middle‐aged samples (|fold change| < 0.2) indicating genuine aging signal with the topmost upregulated and downregulated genes (by fold change) plotted in Figure 1d. A number of these genes have been affiliated with aging such as *Aplnr* (Apelin Receptor) (Rai et al., 2017) and *Ctla4* (Cytotoxic T‐Lymphocyte Associated Protein 4) (Leng et al., 2002) which have been shown to be downregulated and upregulated in aging, respectively, in agreement with our data. Other genes such as *Dlgap2* (Disks large‐associated protein 2), *Galr2* (Galanin Receptor 2), and *Rgs2* (Regulator Of G Protein Signaling 2) have been implicated in age‐related phenotypes including age‐related cognitive decline and body weight gain (Diaz‐Sanchez et al., 2023; Nunn et al., 2011; Ouellette et al., 2020). We then looked for upregulated senescence markers in our previously published mouse bladder single cell data to identify possible SnCs in the mouse bladder (Baker et al., 2021). In single‐cell RNA sequencing (scRNA‐seq) data, no individual cell type or group of cells highly expressed all senescence genes identified by RT‐qPCR and bulk RNA sequencing (Figure 1c). Instead, different cell types appeared to express each gene: *Cdkn2a* (cyclin‐dependent kinase inhibitor 2A, p16) was most highly expressed on a highly differentiated suburothelial fibroblast population identified as highly differentiated myofibroblasts (Baker et al., 2021), with diffuse expression in the urothelium, on immune cells, muscle cells, and endothelial cells (Figure 1c). *Mmp3* (matrix metalloproteinase‐3) was expressed on several fibroblast populations (previously described by our group (Baker et al., 2021)), with diffuse expression on other cell types. *Cxcl1* (C‐X‐C Motif Chemokine Ligand 1, Gro‐α) and *Il6* (Interleukin‐6) had somewhat similar expression on many but not all cell types, however, they didn't coincide with the fibroblast population that is highly enriched for *Cdkn2a* (p16). *Ccl3* (Macrophage inflammatory protein 1‐alpha (MIP‐1‐alpha)) was mostly expressed on immune cells. Finally, *Il10* (interleukin‐10), was expressed on multiple cell types, with the highest expression on immune cells, as was the case for *Tnf* (tumor necrosis factor alpha or TNF‐α) and *Csf2* (Granulocyte‐macrophage colony‐stimulating factor, GM‐CSF) (data not shown). Some urothelial cells appeared to express multiple of these surrogate senescence markers. We hypothesized that, since SnCs represent a very small portion of cells in an organ, yet have a detrimental effect on organ function due to their secretome, that transcriptomics analyses such as those described in Figure 1 may not be sensitive enough to identify them. We therefore proceeded to perform senescence assays using methods that maintained temporal organization of the bladder cells to characterize SnCs in mouse bladders collected over the lifespan (Figure 2). We performed the senescence‐associated β‐galactosidase assay (SA β‐gal) in sections from female and male mice over the lifespan and found that the only SA β‐gal positive cells in the bladder were surface umbrella cells (UCs) lining the lumen (Figure 2a and Figure S7). UCs form a single surface layer and are easily recognizable because they have very large nuclei (2 or more in many species) compared to intermediate urothelial cells due to polyploidy (Wang et al., 2018; Wojcik et al., 2000). Surprisingly, these SA β‐gal positive surface UCs were present throughout the mouse lifespan, as early as 2 months of age, and didn't appear to be aging‐related as previously described for senescent cells in vivo. Whereas the number of SA β‐gal positive cells increased from young to middle age (with maturation), it didn't change with aging (from middle age to old age) (Figure 2b). At 2–6 months, about 30% of luminal urothelial cells were SA β‐gal positive, whereas that number increased to about 45–50% in middle‐aged and old mouse bladders. We observed a similar SA β‐gal staining pattern in male mice over the lifespan (Figure S7). We then looked at another marker of SnCs in mouse bladder sections, the telomere‐associated foci (TAFs). Similarly to what we observed in our SA β‐gal assay, the only cells with 2+ TAFs/cell were surface luminal UCs and there was no change detected in these TAF positive cells over the mouse lifespan (Figures 2f–i and Figures S4 & S5). These cells had very high DNA damage marker γH2AX, which was used to quantify these senescent UCs in whole bladder sections (Figure S5). TAF positive cells represented about 4% of total urothelial cells at all ages tested (Figure 2i, Figure S5). As previously reported, surface luminal UCs in the mouse bladder are also rich in lipofuscin, the so‐called “aging pigment” (Perse et al., 2013), which can be clearly seen in many of the TAF positive cells (Figure 2f). We then looked for *Cdkn2a* (p16) expression in bladder sections by RNA Fluorescence in situ hybridization (RNA FISH, RNAscope). We mostly detected p16 expression in urothelial cells, although this was not restricted to luminal surface UCs as we observed for SA β‐gal positive and TAF positive cells (Figure 2c and Figure S3). We observed a significant increase in percent p16 positive nuclei in the urothelium, increasing from near 0% in middle‐aged mouse bladders to about 10% of total urothelial nuclei of old animals (Figure 2d). These p16‐rich surface UCs were also rich in lipofuscin (Figure S3). To confirm this increase in p16 expression in luminal umbrella cells in mouse bladders with aging, we compared p16 expression in Visium spots (spatial transcriptomics) corresponding to UCs in middle‐aged and old mouse bladders and found p16 expression to be increased in these spots in old relative to middle‐aged bladders (Figure 2e).

To evaluate the transcriptome of superficial UCs over time and see if we could detect any marked differences with aging, we compared gene expression in bladder luminal spots from middle aged and old mice in our Visium spatial transcriptomics data (Baker et al., 2021). We found that middle aged UCs express many anti‐apoptotic genes compared to old UCs (*Hspa8*, *Egr1*, *Hspa1b*, *Hspb1*, *Dnajb1*, *Dnaja1*, *Hspa1a*, *Btg2*, *Cebpb*, and *Gsto1*), whereas old UCs appear to be undergoing a strong stress response suggested by their high mRNA expression of multiple ribosomal protein subunits (*Rpl35*, *Rpl36a*, *Rps4x*, *Rpl30*, *Rpl14*, *Rpl36*, *Rpl38*, *Rpl10*, *Rpl12*, *Rpl13*, *Rpl21*, *Rpl11*, *Rplp2*, *Rps12*, *Rpl23*) shown to play a role in the regulation of the Mdm2/ Mdm4/p53 axis and p53 activation leading to apoptosis, senescence, autophagy, and cell cycle arrest (Kang et al., 2021). The top 20 differentially expressed genes between middle‐aged and old mouse bladder luminal spots are listed in Table S2.

### 2.2 Umbrella cells exhibit protein expression similar to Tetraploidy‐induced senescence

**FIGURE 3:**
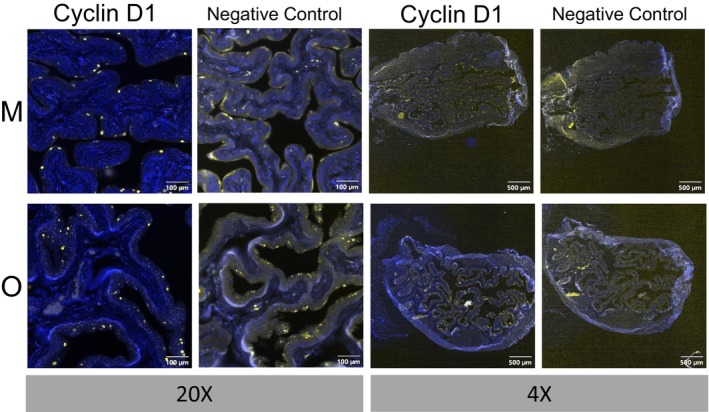
Cyclin D1 staining in Bladder Uroepithelial (Umbrella) Cells. Immunofluorescence staining of Cyclin D1 protein in middle aged (10‐months) and old (26‐months) female mouse bladder sections. Negative controls did not receive primary antibody.

The presence of all markers of senescence we measured in UCs throughout the mouse lifespan and not just with advancing age was unexpected and we wanted to understand the mechanism by which this may be occurring. We looked into the specific biology, structure, and function of UCs to try to explain this observation. Two aspects of UC biology stood out: their barrier function (interfacing with urine), and their polyploidy (having multiple copies of the genome). We hypothesized that the mechanism by which UCs become senescent in vivo may be related to polyploidy (Farsund, 1982; Wojcik et al., 2000). UCs are polyploid (have more than one pair of homologous chromosomes), binucleated and terminally differentiated (postmitotic) cells (Farsund, 1982). Although polyploidy is common in some organisms such as plants, invertebrates, reptiles, and amphibians and during development in mammals, and was traditionally considered a precancerous state in adult mammals, tetraploid, and polyploid cells have recently been described in normal, adult, terminally differentiated cell types in mammalian tissues (bladder, liver, heart, kidney, and eye) (Anatskaya & Vinogradov, 2022). In mammals, polyploid cells can be generated by 3 noncanonical cell cycles in vivo (Zhang et al., 2019). In these cells, polyploidy serves important biological roles; for example, it has been shown to allow cells to better respond to environmental changes or challenges, and repair in addition to protect against oncogenic insults and genotoxic stress (Bailey et al., 2021). It is thought that polyploidy is of particular advantage to facilitate the growth of cells and tissues that need to maintain a barrier because it allows for growth without disruption of cellular connections during cytokinesis (Unhavaithaya & Orr‐Weaver, 2012). In neurons, polyploidization plays a protective role in conditions of DNA damage or oxidative stress (Nandakumar et al., 2021). In short, polyploidy confers stress resistance and increases the lifespan of cells by increasing their resistance to apoptosis, DNA damage and genetic instability, thus allowing their survival in conditions where diploid cells would be unable to survive (Bailey et al., 2021; Ovrebo & Edgar, 2018). A very elegant and thorough study on the cellular mechanisms by which superficial UCs develop and achieve programmed polyploidy in mouse bladders was carried out by Wang et al., who showed that polyploidy arises in bladder UCs via both incomplete cytokinesis and endoreplication and is essential for their barrier function (Wang et al., 2018). However, since polyploidy puts cells at increased risk for aneuploidy and subsequent transformation, the proliferation of polyploid cells is tightly controlled via cell cycle checkpoints in vivo and requires activity of both the p53/p21 and Rb/p16 pathways. Tetraploidy‐induced senescence is a mechanism previously described in vitro in primary rat embryo fibroblasts and human foreskin fibroblasts by Panopoulos et al., 2014. They showed that upon the occurrence of tetraploidy due to cleavage failure in nontransformed primary cells, a non‐DNA‐damage response dependent cell cycle arrest occurs in the G1 phase, with the cells ultimately becoming senescent and expressing SA β‐galactosidase (Panopoulos et al., 2014). This tetraploid arrest, which is dependent on both functional p53 and RB pathways, prevents tetraploid cells from further cell division (Mosieniak & Sikora, 2010; Panopoulos et al., 2014; Rieder & Maiato, 2004). However, cells that are transformed and either lack p16 (Cdkn2a) expression or Rb activity, were able to overcome this tetraploidy‐induced senescent cell arrest. These studies also showed that this premature senescent arrest is not due to increased DNA damage and is instead mediated by a pathway similar to the Ras/Raf oncogene by the cell cycle checkpoint regulator Cyclin D1 (*Ccnd1*) (Panopoulos et al., 2014). To test whether bladder UCs undergo senescence through the Raf‐induced/tetraploidy‐induced senescence pathway, we measured Cyclin D1 protein expression in mouse bladder sections from young, middle‐aged and old mice (Figure 3 [females] and Figure S8 [males]). We found consistently high expression of Cyclin D1 protein strictly in UCs, in a specific pattern mirroring the one we observed for both γH2AX (Figure 2h and Figure S5) and SA β‐gal staining (Figure 2a and Figure S7).

### 2.3 Effect of Senolytics on bladder senescent cells

**FIGURE 4:**
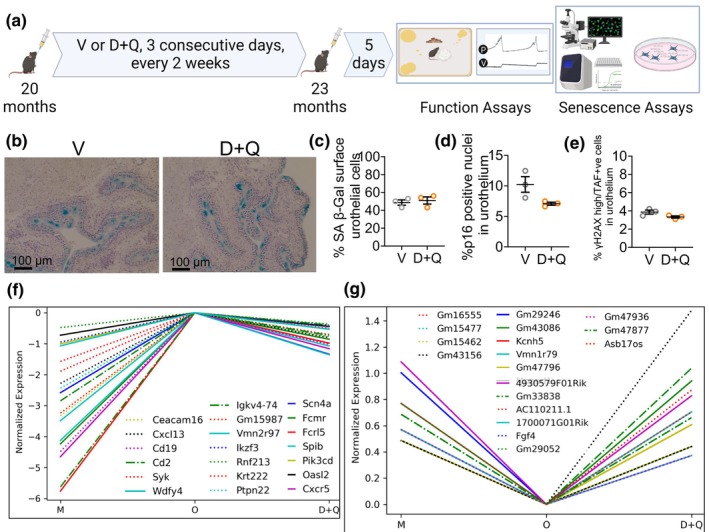
The Effect of D + Q Treatment on Senescence Markers in Old Mouse Bladders. (a) Schematic of senolytics drug treatment regimen used. Created with BioRender.com. (b) Senescence Associated β‐galactosidase assay in old (26‐months) female mouse bladders with Vehicle (V) or Dasatinib+Quercetin (D + Q) treatment. (c) Quantification of Senescence Associated β‐galactosidase assay in old (26‐months) female mouse bladders with V or D + Q treatment. *N* = 3 mice/treatment group. Unpaired two‐tailed *t* test, *significant *p* < 0.05. (d) Quantification of p16 RNAscope in old (26‐months) female mouse bladders with V or D + Q treatment. *N* = 3 mice/treatment group. Unpaired two‐tailed *t* test, *significant *p* < 0.05. (e) Quantification of TAF positive cells in urothelium in old (26‐months) female mouse bladders with V or D + Q treatment. *N* = 3 mice/treatment group. Unpaired two‐tailed *t* test, *significant *p* < 0.05. (f) Whole bladder RNA sequencing data showing most significantly upregulated genes from middle to old age whose expression was decreased by D + Q treatment, normalized to old. (*n* = 4–6 per group, middle aged (10 months), old (26 months) and D + Q‐treated old mouse bladders). Genes plotted are the top 20 significantly increased genes in the middle‐aged versus old analysis (adj. *p*‐value <0.05) that were also significantly decreased in the old versus D + Q analysis (adj. *p*‐value <0.05) after filtering to exclude technical effects (gavage/vehicle treatment). (g) Whole bladder RNA sequencing data showing most significantly downregulated genes from middle to old age whose expression was increased by D + Q treatment, normalized to old. (*n* = 4–6 per group, middle aged (10 months), old (26 months) and D + Q‐treated old mouse bladders). Genes plotted are the top 20 significantly decreased genes in the middle‐aged versus old analysis (adj. *p*‐value <0.05) that were also significantly increased in the old versus D + Q analysis (adj. *p*‐value <0.05) after filtering to exclude technical effects (gavage/vehicle treatment).

Next, we wanted to test whether the senolytic combination of Dasatinib and Quercetin (D + Q) eliminates these senescent UCs in the mouse bladder. D + Q is reported to clear different types of SnCs in many mouse organs and conditions. We treated old mice with Vehicle or D + Q from 20 months to 23 months (Figure 4a) and performed our senescence assays on bladders form these mice. We detected no change in the percentage of SA β‐gal positive UCs after D + Q treatment compared to Vehicle‐treated mice, with that number remaining around 50% (Figure 4b,c). We detected no significant changes in both percentage of TAF positive cells (Figure 4e) and percentage of p16 positive nuclei in the urothelium of bladders from D + Q‐treated mice compared to Vehicle‐treated mice (Figure 4d). Thus, our data show that the senolytic combination D + Q does not ablate senescent UCs in the mouse bladder.

**FIGURE 5:**
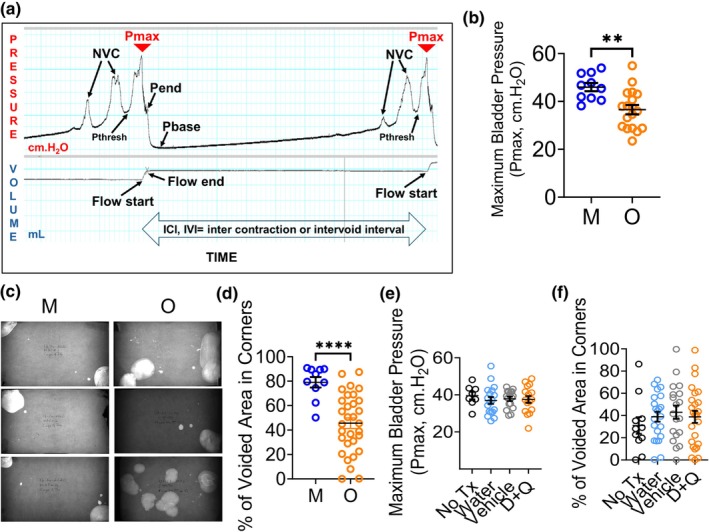
The Effect of D + Q Treatment on Aged Mouse Bladder Function. (a) Urodynamics tracing showing two sequential mouse voids and different parameters measured. NVC = nonvoiding contraction. IVI = intervoid interval. P_max_: Maximum Bladder Pressure, Pbase: Baseline pressure, Pthresh: Threshold pressure, Pend: End of contraction. See Methods for other parameter explanation. (b) Maximum Bladder Pressure (P_max_) difference between middle aged and old female mouse bladders detected by the cystometry protocol used for this study. No changes in other parameters were observed with aging. (*n* = 10 middle aged [M], 18 old [O]). Mean with SEM. Unpaired *T* test with Welch's correction, two‐tailed, *significant *p* < 0.05, **p < 0.005, ***p < 0.0005, ****p < 0.0001. (c) Voiding spot assays (VSA) carried out in middle aged and old female mice. (d) Quantification of VSA showed a significant difference in percentage of voided area in corners between middle aged and old female mice. (*n* = 10 middle aged [M], 31 old [O]). Mean with SEM. Unpaired nonparametric Mann–Whitney *t* test, two‐tailed, *significant *p* < 0.05. (e) Maximum Bladder Pressure (P_max_) measured by cystometry did not change with D + Q treatment. No Tx = no treatment control, Water = water‐gavaged control. Mean with SEM. *N* = 8 no Tx, 18 water, 17 V and 16 D + Q. Ordinary one‐way ANOVA with Dunnett's test for multiple comparisons, compared to vehicle, *significant *p* < 0.05. (f) Percentage of voided area in corners measured by VSA did not change with D + Q treatment. No Tx = no treatment control, Water = water‐gavaged control. Mean with SEM. *N* = 11 no Tx, 22 water, 19 V and 25 D + Q. Ordinary one‐way ANOVA with Dunnett's test for multiple comparisons, compared to vehicle, *significant *p* < 0.05.

Bladder function is controlled by the brain and requires healthy communication between the brain and bladder. Although D + Q did not eliminate bladder SnCs, we wondered whether elimination of SnCs in other parts of the body would contribute to improved bladder function in older mice. To determine whether D + Q treatment causes a positive change in bladder function, we performed two types of bladder functional assays: Voiding Spot Assays (Hill et al., 2018) and urethane‐anesthetized cystometry (Smith & Kuchel, 2010). Using our cystometry protocol optimized to increase the number of old responders, the only difference we observed was a decrease in maximum bladder pressure (P_max_, Figure 5a) with aging in untreated old females compared to untreated middle‐aged females (45 cm.H_2_O (M) versus 37 cm.H_2_O (O), Figure 5b, Table 1). We saw no change in P_max_ in old females following D + Q treatment (Figure 5e, Table 1). In Voiding Spot Assays, the percentage of voided area in corners of untreated old female mice was significantly less than that of untreated middle‐aged female mice (mean of 79% of voided area in corners for middle aged versus mean of 46% of voided area in corners for old mice, Figure 5c,d, Table 2). This aging‐related change in VSA was also not reversed by D + Q treatment in old female mice (Figure 5f, Table 2). Additionally, we performed RNA sequencing of whole bladders from old mice treated with D + Q and found that several genes previously associated with senescence that were increased in the mouse bladders from middle to old age decreased following D + Q treatment, albeit not returning to their “middle‐aged” levels (Figure S6). These include *Cxcr5* (C‐X‐C chemokine receptor type 5), *Cxcl13* (C‐X‐C motif chemokine ligand 13), *Syk* (Spleen tyrosine kinase), and *Pik3cd* (Phosphatidylinositol‐4,5‐Bisphosphate 3‐Kinase Catalytic Subunit Delta) (Bailet et al., 2009; Edwards et al., 2019; Kurosawa et al., 2021; Prudovsky, 2021).

**TABLE 1:** Cystometry Data from All Experiments. Middle‐aged (MA) compared to old (O) female mice; normal chow diet (NCD) compared to high‐fat diet (HFD) fed female mice; HFD‐fed mice compared to HFD‐fed mice gavaged with senolytics (HFD + D + Q); mice intraperitoneally injected with phosphate buffered saline (PBS), nonirradiated mouse ear fibroblasts (Proliferating, Prol.) or irradiated mouse ear fibroblasts (Senescent, Sen.); ungavaged (No Tx), water‐gavaged (Water), vehicle‐gavaged (V) and senolytic‐gavaged old female mice (Dasatinib+Quercetin, D + Q). Parameters measured are baseline pressure (Pbase, cm.H_2_O), threshold pressure before initiation of voiding contraction (Pthresh, cm.H_2_O), maximum bladder pressure reached before voiding starts (P_max_, cm.H_2_O), pressure of voiding contraction ending and sphincter closing (Pend, cm.H_2_O), the time between voids (intervoid interval, IVI, seconds), average voided volume (milliters) and average flow rate (milliliters per second). For each mouse, data is the average of data measured for three consecutive voids. Data presented represents means ± standard deviation (SD).

Variables	Pbase (cm. H_2_O, ±SD)	Pthresh (cm. H_2_O, ±SD)	P_max_ (cm. H_2_O, ±SD)	Pend (cm. H_2_O, ±SD)	IVI (sec, ±SD)	Voided volume (mL, ±SD)	Average flow rate (mL/sec, ±SD)	*n*
Middle‐aged versus Old								
MA	7.03 ± 1.61	16.51 ± 3.85	53.01 ± 4.78	25.32 ± 3.84	172.86 ± 53.86	0.07 ± 0.02	0.03 ± 0.01	10
O	7.46 ± 1.94	20.33 ± 8.17	44.04 ± 8.18	27.67 ± 6.82	199.68 ± 76.47	0.07 ± 0.03	0.03 ± 0.01	18
*p*‐Value	0.5366	0.1052	0.0011*	0.2532	0.2903	0.6563	0.7648	
*Unpaired parametric t test with Welch's correction, two‐tailed*								
High Fat Diet								
NCD	9.89 ± 2.55	22.18 ± 7.73	52.85 ± 5.43	27.96 ± 5.81	106.47 ± 27.60	0.05 ± 0.01	0.02 ± 0.01	8
HFD	9.68 ± 1.55	19.37 ± 3.98	46.26 ± 6.08	26.95 ± 5.22	113.06 ± 30.66	0.04 ± 0.01	0.02 ± 0.02	7
*p*‐Value	0.8446	0.3876	0.0476*	0.7285	0.6712	0.5244	0.7685	
*Unpaired t test, two‐tailed*								
High Fat Diet + Senolytics								
HFD	8.63 ± 1.91	17.26 ± 1.80	42.51 ± 3.96	26.30 ± 4.37	166.45 ± 56.17	0.06 ± 0.01	0.01 ± 0.00	7
HFD + D + Q	10.57 ± 2.74	20.59 ± 4.94	44.84 ± 6.46	28.61 ± 4.21	122.23 ± 49.48	0.04 ± 0.02	0.01 ± 0.01	6
*p*‐Value	0.1812	0.1685	0.4634	0.3536	0.1595	0.1736	0.8861	
*Unpaired t test, two‐tailed*								
Senescent Cell Transplantation								
PBS	5.92 ± 1.47	17.11 ± 2.98	53.04 ± 3.70	21.81 ± 2.74	188.94 ± 64.94	0.07 ± 0.02	0.03 ± 0.01	9
Proliferating	5.84 ± 1.20	15.96 ± 5.30	49.92 ± 6.20	23.05 ± 5.17	181.18 ± 57.11	0.07 ± 0.02	0.03 ± 0.01	10
Senescent	6.48 ± 1.47	16.21 ± 5.39	48.19 ± 3.05	25.25 ± 6.86	188.38 ± 68.14	0.08 ± 0.03	0.03 ± 0.01	11
*p*‐Value (PBS vs. Prol.)	0.9013	0.563	0.1979	0.5196	0.7866	0.9486	0.8305	
*p*‐Value (PBS vs. Sen.)	0.4144	0.6429	0.0063*	0.1514	0.9853	0.5815	0.7574	
*Ordinary one‐way ANOVA, with Dunnett's test for multiple comparisons, compared to PBS*								
Senolytic Treatment								
No Tx	6.02 ± 1.71	16.97 ± 6.74	43.42 ± 6.84	27.67 ± 9.14	172.63 ± 76.16	0.06 ± 0.03	0.02 ± 0.01	8
Water	5.32 ± 1.95	13.95 ± 2.28	41.79 ± 7.04	24.34 ± 6.20	191.30 ± 44.46	0.07 ± 0.02	0.03 ± 0.01	9
V	5.38 ± 2.81	17.66 ± 6.17	43.46 ± 3.89	23.60 ± 6.19	190.77 ± 56.33	0.07 ± 0.02	0.02 ± 0.01	17
D + Q	6.50 ± 2.85	18.11 ± 7.29	43.68 ± 7.51	23.40 ± 4.78	185.35 ± 80.35	0.07 ± 0.03	0.02 ± 0.01	16
*p*‐Value (V vs. No Tx)	0.8506	0.9398	0.7713	0.2062	0.7097	0.9781	0.8506	
*p*‐Value (V vs. Water)	0.983	0.9019	0.9888	0.9265	0.9479	0.8281	0.983	
*p*‐Value (V vs. D + Q)	0.0854	0.9549	0.9743	>0.9999	0.9893	0.9997	0.0854	
*Ordinary one‐way ANOVA, with Dunnett's test for multiple comparisons, compared to Vehicle* (*V*)								

**TABLE 2:** Voiding Spot Assay (VSA) Data from All Experiments. Middle‐aged (MA) compared to old (O) female mice; normal chow diet (NCD) compared to high‐fat diet (HFD) fed female mice; mice intraperitoneally injected with phosphate buffered saline (PBS), nonirradiated mouse ear fibroblasts (Proliferating, Prol.) or irradiated mouse ear fibroblasts (Senescent, Sen.); ungavaged (No Tx), water‐gavaged (Water), vehicle‐gavaged (V) and senolytic‐gavaged old female mice (Dasatinib+Quercetin, D + Q). Data presented represents means ± standard deviation (SD).

Variables	Total number of voids (±SD)	Total void area (cm^2^)	Percent void area in corners (%)	Percentage of large voids of Total voids (%)	*n*
Middle‐aged versus Old					
MA	3.6 ± 1.51	34.73 ± 13.10	79.08 ± 13.52	61.17 ± 30.61	10
O	4.16 ± 2.73	48.8 ± 30.03	45.63 ± 24.25	61.51 ± 31.24	31
*p*‐Value	0.921	0.18	<0.0001	0.995	
*Unpaired non‐parametric Mann‐Whitney t test, two‐tailed*					
High Fat Diet					
NCD	2.2 ± 1.81	47.41 ± 16.35	56.46 ± 23.95	97.14 ± 9.04	10
HFD	2 ± 0.71	41.94 ± 17.31	56.58 ± 24.73	85.19 ± 22.73	9
*p*‐Value	0.711	0.78	0.661	0.141	
*Unpaired non‐parametric Mann‐Whitney t test, two‐tailed*					
Senescent Cell Transplantation					
PBS	2.88 ± 2.19	42.56 ± 21.74	46.22 ± 24.12	74.46 ± 35.12	25
Proliferating	2.35 ± 1.43	42.21 ± 17.43	61.63 ± 15.71	85.35 ± 24.74	23
Senescent	3.55 ± 3.46	40.73 ± 24.08	54.54 ± 26.16	70.38 ± 36.71	22
*p*‐Value (PBS vs. Prol.)	0.6815	0.9976	0.038	0.4129	
*p*‐Value (PBS vs. Sen.)	0.5626	0.9391	0.3494	0.8782	
*Ordinary one‐way ANOVA, with Dunnett's test for multiple comparisons, compared to PBS*					
Senolytic Treatment					
No Tx	3.04 ± 1.57	51.41 ± 35.50	50.29 ± 23.93	75.12 ± 23.03	11
Water	4.18 ± 2.63	47.80 ± 23.60	44.82 ± 19.10	59.36 ± 23.90	22
V	3.79 ± 3.01	50.73 ± 23.36	51.11 ± 19.83	72.82 ± 26.51	19
D + Q	4.06 ± 2.59	49.86 ± 23.51	43.06 ± 26.01	68.50 ± 32.21	25
*p*‐Value (V vs. No Tx)	0.9669	0.7524	0.8537	0.8168	
*p*‐Value (V vs. Water)	0.9107	0.9922	0.6589	0.3265	
*p*‐Value (V vs. D + Q)	0.9857	0.9909	0.6003	0.856	
*Ordinary one‐way ANOVA, with Dunnett's test for multiple comparisons, compared to Vehicle* (*V*)					

### 2.4 Effect of increased SnC burden and systemic inflammation on bladder function

**FIGURE 6:**
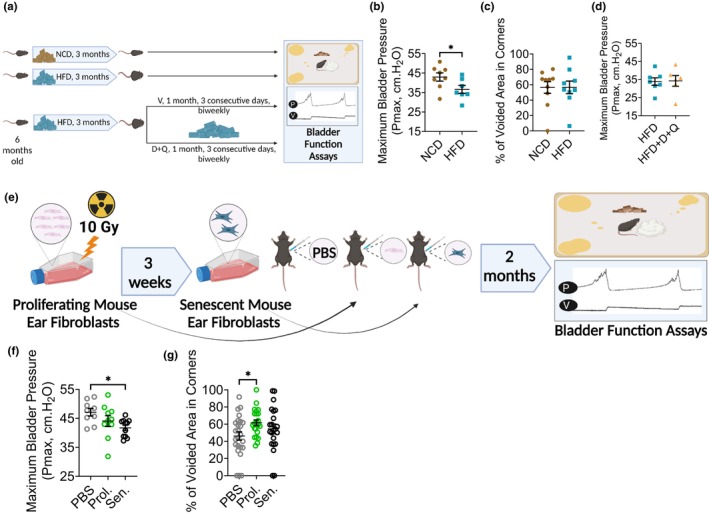
High‐Fat Diet and Senescent Cell Transplantation Induce Bladder Changes Similar to those Observed in Naturally Aged Mice. (a) Top panel: Schematic of High‐Fat Diet (HFD) or Normal Chow Diet (NCD) regimen used; Bottom panel: D + Q treatment regimen during HFD feeding regimen. Created with BioRender.com. (b) Maximum Bladder Pressure (P_max_) difference between NCD‐ and HFD‐fed female mouse bladders detected by cystometry. No changes in other parameters were observed with HFD. (*n* = 8 NCD, 7 HFD). Mean with SEM. Unpaired *t* test, two‐tailed, *significant *p* < 0.05. (c) Voiding spot assays (VSA) carried out in NCD‐ and HFD‐fed female mice showed no difference in percentage of voided area in corners or any other VSA parameters studied. (*n* = 10 NCD, 9 HFD). Mean with SEM. Unpaired *t* test, two‐tailed, *significant *p* < 0.05. (d) Maximum Bladder Pressure (P_max_) difference between HFD‐ and HFD followed by D + Q treatment (HFD + D + Q) in female mouse bladders measured by cystometry. No changes in other cystometric parameters were observed with HFD. (*n* = 7 NCD, 6 HFD). Mean with SEM. Unpaired *t* test, two‐tailed, *significant *p* < 0.05. (e) Schematic of senescent cell preparation and transplantation regimen used. 17‐months old female mice received PBS, Proliferating mouse ear fibroblasts (Prol.) or Senescent mouse ear fibroblasts (Sen.) intraperitoneally and bladder function assays carried out 2 months later. Created with BioRender.com. (f) Maximum Bladder Pressure (P_max_) difference between female mouse bladders detected by the cystometry following IP injection of PBS, Proliferating or Senescent Mouse ear fibroblasts. No changes in other parameters were observed (*n* = 9 PBS, 10 Prol., 11 Sen.). Mean with SEM. Ordinary one‐way ANOVA, with Dunnett's test for multiple comparisons, compared to PBS, *significant *p* < 0.05. (g) Voiding spot assays (VSA) carried out following IP injection of PBS, Proliferating or Senescent Mouse ear fibroblasts showed no difference in percentage of voided area in corners or any other VSA parameters studied (*n* = 25 PBS, 23 Prol., 22 Sen.). Mean with SEM. Ordinary one‐way ANOVA, with Dunnett's test for multiple comparisons, *significant *p* < 0.05.

We then wished to test other models of aging that increase SnC burden and systemic inflammation on mouse bladder function, namely high‐fat diet (HFD) feeding and SnC transplantation. A HFD is believed to increase mouse fat, which increases systemic inflammation and SnC burden because fat has been shown to contain many SnCs (Ogrodnik et al., 2019; Schafer et al., 2016; Wang et al., 2022). We tested the effect of a high‐fat diet on cystometry and voiding spot assay parameters. We fed female C57Bl/6 mice with a normal chow (NCD) or high‐fat diet for 3 months starting at 6 months then performed assays of bladder function (Figure 6a). We found a similar decrease in P_max_ in the HFD group compared to the NCD group as we observed with natural aging (mean P_max_ 43 cm.H_2_O (NCD) versus 37 cm.H_2_O (HFD), Figure 6b, Table 1). Interestingly, we saw no significant change in percent of voided area in corners in the VSA of NCD compared to HFD fed mice as we did for naturally aged mice (Figure 6c, Table 2). This signifies that, although the HFD mimics part of natural aging on age‐related bladder dysfunction, there are other factors at play in natural aging that are detectable by the VSA and not cystometry. This could also reflect a difference between the VSA, for which mice are awake, and the urethane‐anesthetized mouse cystometric assay, where conscious control of bladder function is ablated by urethane anesthesia, thus representing autonomous brainstem reflex to bladder filling instead. We then wished to test whether a short D + Q treatment could reverse the HFD‐induced decrease in P_max_ (Figure 6a, bottom). D + Q had no effect on the HFD‐induced changes in P_max_ (Figure 6d, Table 1).

We additionally tested the effect of SnC transplantation on mouse bladder function (Xu et al., 2018). Mice were intraperitoneally (IP) injected with PBS, proliferating mouse ear fibroblasts or irradiated senescent mouse ear fibroblasts and bladder function assays carried out 2 months later (Figure 6e). As observed for both natural aging and the HFD model, P_max_ decreased significantly in mice injected with SnCs but not in mice injected with proliferating cells (mean P_max_ 47 cm.H_2_O (PBS) versus 42 cm.H_2_O (SEN), Figure 6f, Table 1). There were no significant differences in percentage of voided area in corners between PBS‐ and SnC‐injected mice on VSAs, although a significant but unexplained increase was observed in control mice that received proliferating cells (Figure 6g, Table 2).

## 3 DISCUSSION

Lower urinary tract symptoms and incontinence have a profoundly negative impact on independence and quality of life with aging, with more than one in two older adults confronted with significant symptoms. Nonetheless, current medications typically do not alter the natural history of these debilitating symptoms and are often abandoned due to their frequently troublesome side effects (Gibson et al., 2021). The high prevalence and uniquely complex nature of bladder dysfunction in aging suggests it is a multifactorial geriatric syndrome (Inouye et al., 2007), and therefore geroscience‐guided approaches targeting shared biological pathways of aging may offer potentially transformational therapies (Sierra et al., 2021).

Senescent cells have been identified and studied in most organs in mice and humans, including the brain, heart, lungs, kidneys, liver, pancreas, skin, and eyes (Suryadevara et al., 2024; Tuttle et al., 2020). They accumulate with aging and are often present at sites of disease such as in lungs of chronic obstructive pulmonary disease (COPD) and Idiopathic pulmonary fibrosis (IPF) patients or the kidneys of diabetics with kidney disease (Suryadevara et al., 2024). Clearing SnCs in vivo using senolytics represents one of the most promising efforts at targeting biological pathways of aging shared by varied chronic diseases and geriatric syndromes. This approach has now been shown to delay or reverse several aging phenotypes in preclinical models, and clinical trials testing senolytics to improve a myriad of chronic diseases and geriatric syndromes (e.g., frailty) are underway (Gasek et al., 2021). However, several beneficial, physiological roles for senescent cells have also been described (de Magalhaes, 2024).

To our knowledge, this study represents the first comprehensive study of SnCs in the bladder showing umbrella cell (UC) senescence over the mouse lifespan. Normal human uroepithelial cells (HUCs) have previously been shown to undergo replicative senescence in vitro via p16INK4A, with arrest occurring in the G1/S phase of the cell cycle (Puthenveettil et al., 1999). HUCs were also found to undergo senescence in vitro in response to low‐dose intermittent H_2_O_2_ exposure, but this growth arrest appeared to occur in the G2/M phase of the cell cycle in a p16INK4A‐independent mechanism (Chien et al., 2000). Klee et al. reported increased SA β‐galactosidase staining in urothelial cells of 53‐week‐old Wistar rats and in rats where diabetic bladder dysfunction (DBD) was induced with a combination of HFD and streptozotocin injection relative to control young (20‐week old) and nondiabetic animals (Klee et al., 2018). In contrast to our findings across the lifespan (Joshi et al., 2024), Joshi et al. had recently reported SA β‐gal and γH2AX urothelial staining but limited to urothelium in old female mice (Joshi et al., 2024). As recently summarized by NIH Cellular Senescence Network (SenNeT), such discrepancy can be attributed to the very low specificity of SA β‐gal staining with frequent false positives resulting from suboptimal techniques and analysis (Suryadevara et al., 2024). Also, great care must be taken to distinguish fluorescence reflective of immunohistochemical signal from that indicating endogenous autofluorescent lipofuscin droplets (Figure 2f) which accumulate in nearly all tissues with aging (Suryadevara et al., 2024). Moreover, while γH2AX staining must be by definition nuclear, such staining reported by Joshi et al. in UCs appeared strictly cytoplasmic (Joshi et al., 2024).

Our study revealed that umbrella cells (UCs), the terminally differentiated surface urothelial cells in the bladder that line the bladder lumen, exhibit multiple characteristics of SnCs and may thus represent a senescent cell population in the bladder. Interestingly, this population of SnCs is different from other aging‐related SnCs as it is present from a young age and throughout the adult lifespan into old age. We attribute this novel observation to the interesting biology of bladder UCs (Dalghi et al., 2020), whose function is to form a barrier to protect the bladder and organism from pathogens and the harsh chemical composition of urine, preventing them from seeping into the bladder wall and entering the circulation. UCs are a very long‐lived (up to 200 days in rodents), terminally differentiated type of epithelial cell forming a single layer at the interface of the urine in the bladder lumen and underlying bladder tissue. They are joined by tight junctions, covered in plaques and a mucin layer on their apical membrane, all working together to ensure impermeability. Another means by which the impermeability of the bladder‐urine interface is maintained is through polyploidy of UCs, which increases the lifespan of these cells by conferring stress resistance, thus allowing their survival in conditions where diploid cells would be unable to survive (Bailey et al., 2021; Ovrebo & Edgar, 2018; Wang et al., 2018). Furthermore, polyploidy serves several important biological roles; (1) it allows cells to better respond to environmental changes or challenges, and repair (Bailey et al., 2021); (2) it protects against oncogenic insults and genotoxic stress; and (3) it facilitate the growth of cells and tissues that make up biological barriers, because it allows for growth without disruption of cellular connections by cytokinesis (Unhavaithaya & Orr‐Weaver, 2012; Wang et al., 2018). Polyploidy in the bladder UCs results through the biologically programmed processes of incomplete cytokinesis and endoreplication (Wang et al., 2018). Approximately one third of superficial UCs, but not underlying uroepithelial cells, obtained from bladder washes from healthy people whose bladders were free from cancer and whose specimens had no evidence of malignancy or dysplasia, were tetraploid (Wojcik et al., 2000). Additionally, 45% of people with tetraploid DNA content in bladder washes had no clinical or pathologic evidence of tumors (Kline et al., 1995). Our data showing strong cyclin D1 protein expression specifically in UCs in male and female mice throughout the lifespan, support that the Raf‐induced senescence‐like tetraploidy‐induced senescence described in tetraploid cells in vitro, also exists in vivo in these bladder UCs that also exhibit several other markers of cellular senescence. To our knowledge, this is the first example of in vivo tetraploidy/polyploidy‐induced senescence in terminally differentiated cells in adult organisms. Based on this finding of a novel beneficial role for cellular senescence in vivo, we postulated the presence of other barrier cells in vivo exhibiting polyploidy‐induced senescence throughout the lifespan, in a nonaging‐related manner. Similarly, an essential role for vascular endothelial senescent cells as blood‐tissue barrier cells, especially liver sinusoid endothelial cells (LSECs) has been described in vivo (Grosse et al., 2020). Genetic clearance of senescent endothelial barrier cells in vivo over the mouse lifespan led to liver and perivascular tissue fibrosis and illness in these mice by middle age (Grosse et al., 2020). These senescent endothelial barrier cells were identified in livers, hearts, lungs, and kidneys and could not be efficiently replaced in vivo after genetic ablation. Our data present the first description of the role of cellular senescence in barrier function in the bladder and maintenance of the urine‐bladder barrier.

Because they are very long‐lived and constantly exposed to the urine and its toxic content, UCs have many lysosomes to help clear waste. This higher‐than‐normal lysosomal content and activity likely contribute to the SA β‐galactosidase activity observed in these cells throughout the lifespan. Furthermore, this enhanced lysosomal activity can protect these UCs from oxidative stress and possibly contributes to their resistance to apoptosis (Chakraborty et al., 2019; Li et al., 2021). It has been recently reported that the function of lysosomes in the mouse bladder urothelium is impaired in middle aged (15–18 months old) mice, leading to their accumulation (Joshi et al., 2024). These UC characteristics can also explain the accumulation of lipofuscin in these cells, which has previously been reported (Perse et al., 2013) and observed in our study to increase with aging. Lipofuscin is often present in the cytosol of old cells; it is made of proteins and lipids damaged by free radicals. Cells cannot degrade lipofuscin and it can lead to mitochondrial dysfunction (Jung et al., 2007).

The fact that these UCs appear senescent from a young age, however, doesn't mean that young UCs are physiologically identical to old UCs, or that UCs from aged animals are as healthy as those from young ones. Our spatial transcriptomics data showing a deregulation in transcription of ribosomal subunits with aging in luminal spots is suggestive of increased stress in these cells with aging. Moreover, Perse et al., 2013 showed that UCs in old mice had increased oxidative stress markers such as malondialdehyde (MDA) and inducible nitric oxide synthase (iNOS), impaired total antioxidant capacity and severe mitochondrial dysfunction. They wondered whether these cells experiencing so much oxidative stress undergo increased apoptosis in older animals but didn't find it to be the case. They argue that “superficial urothelial cells have a strong compensatory mechanism to counteract age‐related changes” and that “this protective mechanism against oxidative changes is actually to be expected, since superficial cells are the main component of the blood‐urine barrier.” Perse et al., 2013 also found no increase in proliferation, as evidenced by no increase in Ki67 staining in old mouse umbrella cells compared to young mice (Perse et al., 2013), which occurs in intermediate uroepithelial cells after urothelial injury or infection to replace lost umbrella cells (Wang et al., 2018); they also reported no increased apoptosis of uroepithelial cells in old mouse urothelium by caspase 3 staining. Combined with our senescence data, we now believe this mechanism they were referring to is cellular senescence.

Our in vivo study with D + Q has some limitations. It has been reported that systemic administration of drugs to treat bladder conditions is often ineffective because only a small fraction of the drug reaches its target (GuhaSarkar & Banerjee, 2010), due to the low perfusion of the urinary bladder. Although we did see transcriptomic changes in RNA sequencing experiments that provide evidence that D + Q was having an effect in the bladder, systemic drug delivery to the UCs may be particularly difficult, as the urothelial layer is poorly perfused. This fact may explain the failure of D + Q to eliminate senescent bladder UCs in our assays, even though we observed and reported benefits of our D + Q treatment regimen and methodology in other systems, such as a positive effect on preserving mandibular condylar cartilage thickness, improving subchondral bone volume and turnover, and reducing Osteoarthritis Research Society International (OARSI) histopathological score in old mice (Zhou et al., 2021). Direct infusion of drugs into the bladder and different intravesical delivery methods are being developed for targeting different bladder conditions such as bladder cancer, interstitial cystitis, and infections. Translationally, this finding is important because it suggests that D + Q given orally might not deplete this important barrier when used in humans, thus not compromising the blood‐urine barrier. Another reason our treatment may have failed to get rid of senescent UCs in the mouse bladder is that, although the combination of D + Q kills a broad range of SnCs, this combination may not target epithelial cell types, which bladder UCs belong to (Palmer et al., 2019; Zhu et al., 2015). No known senolytic to date kills all types of SnCs. Additionally, since tetraploidy‐induced senescence uses different mechanisms for apoptosis resistance and cell cycle arrest, these SnCs may not be sensitive to senolytics that kill SnCs induced through other pathways, such as replicative senescence or stress‐induced senescence, which may upregulate other Senescent Cell Anti‐Apoptotic Pathways (SCAPs) (Kirkland & Tchkonia, 2017). Similar to our findings, D + Q oral gavage of mice did not remove senescent endothelial barrier cells from mouse livers, although it decreased the number of F4/80‐positive macrophages, showing that D + Q was also inactive and safe on these senescent endothelial barrier cells (Grosse et al., 2020). We therefore propose that since “killing” bladder UCs and other potentially senescent barrier cells may compromise their barrier function causing harm to the organ and organism, treatments that improve the health of these senescent barrier cells, such as decreasing their oxidative stress or improving their mitochondrial health, may be safer and more beneficial than clearing them. Our data support the notion that not all senescent cells are pathological and thus not all senescent cells should be cleared in vivo (Grosse et al., 2020).

Finally, we hypothesize that our findings may have implications in further elucidating the biology of some bladder cancers. Urothelial carcinoma, previously called transitional cell carcinoma, is the most common type of bladder cancer in the United States. It can occur anywhere in the urinary system, such as in the renal pelvis, ureters, bladder, and urethra, but is most commonly found in the bladder. One of the main roles of SnCs is believed to be the prevention of malignant transformation by halting cell division in proliferating cells that have accumulated molecular damage (including telomere shortening) or where oncogenic activation (e.g., H‐RASV^1269^) occurred (Campisi, 2013; Schmitt et al., 2022; Serrano et al., 1997). In the latter case, senescence can be induced by robust, persistent, or unbalanced mitogenic signals (Zhu et al., 1998). Although the oncogenes Ras and Raf cause the transformation of immortalized cells, their activation in primary cells causes them to enter premature senescence instead (Zhu et al., 1998). In these oncogene‐induced senescent cells, there is increased Cyclin D1 protein production (Zhu et al., 1998). We observed specific and robust Cyclin D1 expression in mouse UCs over the lifespan. Based on our data and those published about in vitro tetraploidy‐induced senescence, activation of the oncogene Cyclin D1 in response to tetraploidy may play a role in the senescent arrest of superficial UCs (Panopoulos et al., 2014; Zhu et al., 1998). The ability of cells to arrest in G1 when tetraploidy is induced, is important for controlling tumor growth, since it represents the last chance for tetraploid cells to avoid aneuploidy. Cyclin D1 is an important cell cycle regulator that modulates the transition from G1 to S phase. Its expression is found to be upregulated in many cancer types, including breast cancer, lung cancer, and melanoma, and is often used as a biomarker for bladder cancer (Tchakarska & Sola, 2020). Overexpression of Cyclin D1 alone, however, does not lead to cancer; instead, a concurrent inactivation or loss of p53/21 or p16/Rb or, alternatively, activation of a second oncogene, appears to take place prior to cell transformation, with both an intact p53 and Retinoblastoma pathways essential for maintaining this senescent cell arrest of tetraploid cells (Hosokawa et al., 2001; Montalto & De Amicis, 2020; Panopoulos et al., 2014; Serrano et al., 1997; Zhu et al., 1998). Coincidentally, loss of p16 expression in urothelial cells found in urine is a sensitive and specific clinically used biomarker for detection of urothelial cancers, and chromosome 9 deletion (where *Cdkn2a* resides) is one of the most frequent mutations associated with urothelial bladder cancer (Orlow et al., 1995; Wu et al., 2000). These data, in conjunction with the loss of p16 expression in many cancers (bladder and others), and the observation that many cancers have abnormally high chromosome numbers but few mutations, strongly suggests that dysregulation of senescent arrest in bladder superficial UCs leads to their aneuploidy, resulting in transformation (Gordon et al., 2012). Therefore, levels and activities of Cyclin D1, p16, p21, p53, and Rb, all have to be tightly regulated to maintain the superficial UCs in a senescent, permanently arrested state. However, if this permanent cell arrest is disturbed, polyploid cells are at a higher risk of becoming aneuploid by undergoing further aberrant divisions that result in chromosomal instability and generate cancers (Ganem & Pellman, 2007; Gordon et al., 2012). This is in agreement with and further supports the hypothesis that cancer cells are cells that escaped senescent cell arrest (Campisi, 2013; Schmitt et al., 2022). Factors such as smoking and frequent urinary tract infections could act as precipitating factors on an already stressed background of senescent UCs, causing that “second hit” required for aneuploidy and transformation. Additionally, the ribosomal stress response we observed in our transcriptomics data has been linked to an increased susceptibility to cancer, as ribosomal proteins have been shown to gain oncogenic potential and lead to transformation (Kang et al., 2021). Moreover, it has been shown that, through their secretome (the SASP), SnCs can cause malignant transformation of surrounding cells. It is conceivable that, whereas senescence of UCs arose to maintain the blood‐urine barrier as herein postulated, which is essential for organismal survival and fitness earlier in life, they sometimes cause malignant transformation of urothelial cells in their vicinity, resulting in urothelial carcinomas. Finally, differential expression between UCs of middle aged and old mice shows striking changes in expression of ribosomal proteins in old UCs relative to middle aged UCs, which are increasingly linked to susceptibility to cancer (Kang et al., 2021). This scenario represents another way that SnCs are antagonistically pleiotropic, providing an advantage early in life, with potential future detrimental effects.

We used continuous uroflow cystometry to assess bladder function in a pressure/flow multichannel urethane‐anesthetized mouse cystometry model for our studies (Smith & Kuchel, 2010). We observed that old mice needed a longer recovery period following catheter implantation surgery (45–60 min) compared to young or middle‐aged animals to begin cycling in response to bladder filling. In order to obtain urodynamics data from all experimental groups and decrease the previously reported nonresponder rate of about 50%–66% in old female C57BL/6 mice, all mice were allowed a longer recovery period (45 min) after the catheter implantation procedure (Fraser et al., 2020). Following this recovery period, most old mice responded to the physiologic challenge of cystometry, thus enabling us to collect and obtain cystometric data. We also found that older mice took longer to establish a uniform reflex voiding pattern than young mice, however, once they did, we only observed differences in maximum bladder pressure between middle‐aged and old mice, with no differences in inter‐void intervals, per‐void volumes, and voiding flow rates. This represents an example of decreased resilience to stressors with aging, as the older animals took longer to recover from anesthesia (isoflurane) and surgery and establish a uniform reflex voiding pattern to continuous bladder filling (Fraser et al., 2020).

Although the HFD model has previously been used in multiple mouse and rat strains to induce bladder dysfunction, SnC transplantation has never been shown to induce bladder changes. Using continuous pressure/flow multichannel urethane‐anesthetized cystometry, we saw a decrease in maximum bladder pressure in both HFD‐fed and SnC‐transplanted mouse models, mimicking what we observed with natural aging in old mice. This further supports the hypothesis that systemic inflammation, regardless of initiating factor, plays a role in the pathophysiology of bladder dysfunction (Klee et al., 2018). This decrease in maximum bladder pressure during reflex voiding may be caused by changes in the bladder (e.g., changes to extracellular matrix and fibrosis, altered signaling, smooth muscle dysfunction, etc.) or the urethra (i.e., altered signaling to urethral sphincter, smooth and/or skeletal muscle dysfunction, etc.) or a combination of both. Translationally, opening of the external urethral sphincter and voiding at a lower pressure could translate into leaking (incontinence), which is often seen in humans living with metabolic syndrome and obesity. However, more experiments would need to be done to confirm this hypothesis. It is worth noting that rodent cystometry methods are variable and outcomes are dependent on the specific methods used. In addition to mouse sex and strain, factors such as awake versus anesthetized cystometry, the recovery time between catheter placement surgery and cystometry, size of the catheter used, length of saline infusion before data collection, type of anesthesia, rate of saline infusion, all have an effect on cystometric parameters, making comparisons between different studies difficult (Bjorling et al., 2015; Fraser et al., 2020). Data interpretation needs to be done both in the context of the animal model used and the specific functional assays employed. Different cystometry methods or voiding spot assays may provide complementary explanation to questions of bladder dysfunction in rodents.

In addition to cystometry, the voiding spot assay represents a useful assay for assessing bladder function, with clear differences in voiding patterns between young/mature and old mice with bladder dysfunction. Our VSA data suggest that, although the HFD mimics part of natural aging on age‐related bladder dysfunction, there are other factors at play in natural aging that are detectable by the VSA and not cystometry. This could also reflect a difference between the VSA, for which mice are awake, and the urethane‐anesthetized mouse cystometric assay, where conscious control of bladder function is ablated by urethane anesthesia, thus representing autonomous brainstem reflex to bladder filling. Care must be taken when performing the mouse VSA to ensure factors that can affect mouse voiding behavior (such as time of day when assays are done), are well controlled for and reported (Bjorling et al., 2015; Chen et al., 2017; Hill et al., 2018; Luo et al., 2023). Since female mice do not exhibit marking behavior as male mice do, this assay may be more useful in female than male mice.

Although striking, the sex differences we observed in our transcriptomics data were not surprising and there are many known differences in LUT function between males and females; for example, the time course of LUT dysfunction is more rapid in females than in males. It is increasingly clear that sex differences exist between male and female bladders, with female bladders having more inflammatory phenotypes and more upregulated innate immunity and tissue remodeling pathways than males (Peskar et al., 2023). This may explain the higher rate of occurrence of many bladder diseases including interstitial cystitis, overactive bladder syndrome, and recurrent urinary tract infections in women (Maserejian et al., 2013). Women may therefore benefit from therapies that target the root of this bladder inflammation that can manifest in various diseases and symptoms. However, the senescence of bladder umbrella cells appears to be conserved across the sexes.

## 4 EXPERIMENTAL PROCEDURES

### 4.1 Mice

Studies were conducted in C57BL/6 mice received from the NIA aging mouse colony. Unless otherwise specified, mouse ages were: young (2–4 months), middle aged (10–12 months) and old (20–26 months). Mice were group housed (5/cage) in a climate‐controlled environment, fed standard rodent chow (except for high‐fat diet study) and water ad libitum, with a 12/12 dark/light cycle. All mice were cared for in accordance with the recommendations in the Guide for the Care and use of Laboratory Animals of the National Institutes of Health. All procedures were approved by the University Of Connecticut School Of Medicine/UConn Health institutional animal care and use committee (IACUC), to PPS (TE‐101873‐0621, AP‐200404‐0324) and MX (TE‐101819‐0421).

### 4.2 Senolytic treatment

Mice were treated with either the senolytic combination D + Q (5 mg/kg/day dasatinib [D33071G, LC Laboratories] and 50 mg/kg/day quercetin [Q4951‐10G, Sigma‐Aldrich]) or vehicle control 60% Phosal 50 PG (368,315 LIPOID LLC), 30% polyethylene glycol 400 (PEG‐400 Bio‐Ultra, 91,893‐250ML‐F), Sigma‐Aldrich, 10% ethanol (BP2818500, Fisher BioReagents, Fisher Scientific) as previously described (Palmer et al., 2019; Xu et al., 2018; Zhou et al., 2021). D + Q were dissolved in vehicle at a concentration of 2 mg/mL and 20 mg/mL, respectively. D + Q/V was orally gavaged; several treatments consisting of three consecutive days separated either by two (mice treated from 20 to 23 mo.) or four (mice treated from 21 to 26 mo.) weeks. Mice were allowed to rest for 5 days prior to use in experiments.

### 4.3 RT‐qPCR

Bladders (*n* = 4–8 per age/sex) from young, middle aged and old male and female mice were harvested into RNAlater (Qiagen), kept overnight at 4°C before freezing at −20°C. RNA extraction was done using the Nucleospin RNA isolation kit (Clonetech) per manufacturer's protocol. Bladders were placed in 350 μL lysis buffer in a 2 mL 2.8 mm ceramic bead containing microtube and homogenized using a Fisherbrand beadmill 24 bead homogenizer (speed 6, 2 cycles, 10 s per cycle, 1 min rest between cycles). RNA quality and concentration were determined by Nanodrop 2000c (Thermo Scientific, Waltham, MA). Equal amounts of RNA were reverse transcribed to cDNA using the iScript™ Reverse Transcription Supermix (Bio‐Rad Laboratories, Inc.) per manufacturer protocol. One hundred nanograms cDNA was used per qRT‐PCR reaction, using the SsoAdvanced™ Universal SYBR® Green Supermix (Bio‐Rad Laboratories, Inc.) and predesigned Biorad PrimePCR™ SYBR® Green Assays for mouse genes for detecting senescence and SASP (Table S1). *Rps18*, *Yhwaz*, and *B2m*, showed the least variability between young and old bladders and thus were determined to be suitable reference genes in a screen of 14 commonly used reference genes (data not shown). PCR, gDNA, RT and RNA quality controls were all run simultaneously. Gene expression was calculated via a modified Pfaffl method utilizing multiple reference genes (Pfaffl, 2001; Vandesompele et al., 2002). Data were normalized to gene expression of young sex‐matched mice to give comparable fold changes by geometric averaging of three internal control genes.

### 4.4 RNA sequencing

Bladders (*n* = 4–6/group) from young (2 mo.), middle aged (10 mo.), old (26 mo.), water‐treated old, vehicle‐treated old and D + Q‐treated old (26 mo., treated from 21 to 26 mo. monthly) C57BL/6 female mice were harvested into RNAlater (Qiagen). RNA was extracted as described above (RT‐qPCR). Samples were submitted to the UConn Institute for Systems Genomics where library preparation for total RNA‐Sequencing was carried out using the Illumina TruSeq Stranded Total RNA library preparation kit followed by High Output Illumina Sequencing on the Illumina NextSeq 500/550 sequencer using the v2.5 150 cycle reagent kit (Estimated total reads per sample = 180–200 M 150 bp paired end reads). Fastq files were aligned via Star (2.7.3) to the mouse mm10 reference (mm10‐2020‐A). Gene counts were quantified via subread featureCounts (2.0) and used as input for differential expression analysis with edgeR (3.28.1). Normalized expression for plotting was done via transcripts per million (TPM) normalization. To account for technical effects (e.g., gavage), differential expression was performed between water only, vehicle only, D + Q treated and untreated samples from the same age (26 months). Resultant differentially expressed gene lists from the D + Q treated and untreated filtered to exclude those found to be significant in water only and vehicle only analyses to isolate effects of D + Q (|fold change| in water only/vehicle only vs. untreated <0.2). In a similar fashion, to determine genes which are uniquely affected by aging, genes found to be significantly differentially expressed between old and middle‐aged samples (adj. *p* value <0.05) were filtered to remove those genes found to be significantly differentially expressed between young and middle aged samples (|fold change| <0.2). Genes selected for plotting were those filtered significant genes of highest |fold change| in old versus middle aged analysis.

### 4.5 RNAscope

RNA FISH for p16 was carried out on bladders obtained from middle aged, untreated old, vehicle‐treated old and D + Q‐treated old female mice (26 mo., treated monthly from 21 to 26 mo., 3 consecutive days/treatment). Mice (*n* = 3 old, *n* = 2 middle aged) were euthanized by CO_2_ asphyxiation and bladders immediately excised and placed in 10%NBF at room temperature for 18–24 h on a shaker. Following 3 one‐hour washes with cold PBS at 4°C, bladders were dehydrated in cold 50% ethanol (twice, 30 min each) followed by 70% ethanol (twice, 30 min each). Bladders were kept overnight in 70% ethanol at 4°C and embedded in paraffin the following day. Bladder sections (6 μm) were cut onto Tissue Path Superfrost Plus Gold microscope slides (Fisherbrand 15–188‐48). RNA in situ hybridization experiments were performed using the RNAscope® technology, which has been previously described (Wang et al., 2012). RNAscope was carried out following manufacturer protocol for FFPE samples using the RNAscope Multiplex Fluorescent Reagent Kit V2 (Advanced Cell Diagnostics INC, Newark, CA). Standard tissue pretreatment (target retrieval (15 min) and protease digestion (30 min)) conditions were used. RNAscope probes used were as follows: smooth muscle actin (Mm‐Acta2‐C1, 319,531) and p16 (Mm‐Cdkn2a‐C2, 411,011‐C2). Each sample was quality controlled for RNA integrity with a probe specific to the housekeeping gene PPIB (Peptidylprolyl Isomerase B). Negative control background staining was evaluated using a probe specific to the bacterial dapB (L‐2,3‐dihydrodipicolinate reductase) gene. For Channel 1, Opal520 dye was used at a 1:1500 dilution, for Channel 2, Opal690 was used at a 1:750 dilution (FP1487001KT, FP1497001KT, Akoya Biosciences Inc., Marlborough, MA). Slides were imaged at 20X (20x/0.8 Plan‐Apochromat objective) and 40X (40x/1.3 Achrostigmat Oil objective) on a ZEISS LSM 880 confocal microscope mounted on an Axio Observer Z1 using FITC filter settings for Opal520 and Cy5 filter settings for Opal690. Images were analyzed using CellProfiler for automated counting. Object thresholds were defined in separate fluorescent channels according to 10th percentile of parallel experimental positive control. This allowed for minimizing risk of false positives but still capture faint puncta signal relative to background (negative control). Pixel diameters were established based on 10th to 90th percentile using CellProfiler histogram feature for all channels. DAPI‐positivity was used to localize p16 signal within defined nucleus parameters (10–50 pixel units). We used a 3–10 pixel unit diameter to define p16 punctae to exclude signal from debris or other objects (ie. lipofuscin). The same analysis was conducted in parallel with actin (3–50 pixel units). Total samples analyzed included three biologic replicates per group (except middle aged where *n* = 2), three sections per animal, and three regions of interest (ROI, detrusor (DSM) versus urothelial (URO) layers) per section. Exported image data included total p16‐positive puncta in image field, total p‐16 positive cells in field, total actin‐positive cells in field, and co‐expressing p‐16 positive, actin‐positive cells in field. Data were analyzed by an unpaired, two‐tailed *t* test with Welch's correction. For comparing of Vehicle and D + Q‐treatment we used an unpaired, two‐tailed *t* test (*p* < 0.05, significant). Statistics done in GraphPad Prism 9.0.

### 4.6 Senescence‐associated β‐galactosidase assay (SA β‐gal)

SA β‐gal staining was carried out on bladders (*n* = 3 per group) obtained from young, middle aged, old (26 months), vehicle‐treated old and D + Q‐treated old female mice (26 mo., treated monthly from 21 to 26 mo., 3 consecutive days/treatment). Mice were euthanized by CO_2_ asphyxiation and bladders immediately excised, placed in an OCT‐filled mold and frozen in liquid nitrogen‐cooled isopentane and kept on dry ice until cutting on the same day. Sections (6 μm) were cut on a cryostat and kept on dry ice until fixing (cold, freshly prepared fixative [2% formaldehyde and 0.25% glutaraldehyde in PBS]) for 15 min at RT. Following two 10‐min PBS washes, sections were placed in freshly prepared SA β‐gal staining solution (1 mg/mL X‐gal, 40 mM citric acid/sodium phosphate buffer, 5 mM potassium ferrocyanide, 5 mM potassium ferricyanide, 150 mM sodium chloride, 2 mM magnesium chloride, pH 6.0) and incubated in a humidified incubator without CO_2_ (18 h). Slides were washed with PBS, and nuclei counterstained with Hematoxylin, dried in ethanol and xylene before coverslipping. Images were taken using brightfield microscopy at 4x and 20X. A total of 3 fields were counted per section and three sections were analyzed per bladder. Data are represented as percentage SA‐β‐gal‐positive (blue) cells relative to the total number of luminal urothelial cells. For the 3‐age group analysis, we used an ordinary one‐way ANOVA, with Tukey test for multiple comparisons. For comparing of Vehicle versus D + Q‐treatment we used an unpaired, two‐tailed *t* test (*p* < 0.05 was considered significant). Statistics done in GraphPad Prism 9.0. Male lifespan SA β‐gal staining results (*n* = 3 per group) are also shown in Figure S7.

### 4.7 Single cell RNA sequencing (scRNA‐seq) and spatial transcriptomics

Visium spatial transcriptomics and scRNA‐seq data was used from GSE180128 (Baker et al., 2021). Uniform Manifold Approximation and Projection (UMAP) embeddings, cellular annotations and gene expression were used from the original scRNA‐seq datasets in order to map significant genes identified in bulk RNA seq onto cell types. Example genes for plotting were chosen as those genes which were significant based on bulk expression (adj. *p*‐value <0.05) and which were present in at least one cell type specific gene list from GSE180128 (Baker et al., 2021). Expression of *Cdkn2a* in luminal urothelial cells was performed by isolating luminal cells identified in Baker et al., 2021 and performing a Wilcoxon rank sum test of *Cdkn2a* expression between middle aged and old bladders (*n* = 4).

### 4.8 Telomere‐associated foci (TAF)

TAF staining was carried out on bladders (*n* = 3 per group) obtained from young, middle aged, untreated old, vehicle‐treated old and D + Q‐treated old female mice (26 mo., treated monthly from 21 to 26 mo., 3 consecutive days/treatment). Mice were euthanized by CO_2_ asphyxiation, bladders immediately excised and processed for paraffin embedding and sectioning (6 μm) as described above for RNAscope. TAF assays were carried out as previously described (Xu et al., 2018). Briefly, sections were dewaxed in Histoclear (National Diagnostics, Charlotte, NC), hydrated with a decreasing ethanol gradient, washed with water, and heat‐mediated antigen‐retrieval carried out in 0.1 M citrate buffer, pH 6.0. Slides were blocked for 1 h in 1:60 goat serum (NGS) in 0.1%BSA in PBS then further blocked with avidin/biotin blocking (NC9406552, Vector laboratories) for 15 min at RT. Primary antibody (1:250 anti‐γH2AX Phospho‐Histone H2A.X (Ser139) (20E3) Rabbit mAb, cat. no. 9718S, Cell Signaling diluted in 1:60NGS/0.1%BSA in PBS) was added and incubated overnight at 4°C in a humidified chamber. After washing in PBS, secondary antibody (1:200 biotinylated Goat Anti‐Rabbit IgG Antibody (BA‐1000, Vector laboratories) diluted in 1:60NGS/0.1%BSA in PBS) was applied and incubated for 1 h at RT. After PBS washes, Dylight649‐conjugated streptavidin (1:500 in PBS, SA‐5649‐1) was applied to sections and incubated for 20 min at RT. Samples were crosslinked with 4% paraformaldehyde in PBS for 20 min at RT. Sections were washed in PBS and dehydrated using an ethanol gradient (3 min each in ice cold 70%, 90% and 100% ethanol). After air drying, hybridization mix (70% deionized formamide, 1 mM Tris–HCl pH 7.2, 25 mM Magnesium Chloride, 1ug/mL Cy‐3 CCCTAA probe, 5% Roche blocking buffer) was added to each section and covered with coverslips. Sections were placed in an oven at 82°C for 10 min to denature DNA, then placed in a humidified chamber in the dark for 2 h at RT to hybridize. Sections were then washed once with 70%formamide in 2x SSC buffer, washed twice with 2X SSC, and finally washed once in PBS (10‐min/wash). Sections were mounted in ProLong™ Glass Antifade Mountant with NucBlue™ Stain (Invitrogen, P36981) and allowed to sit overnight in the dark before sealing and placing at 4°C. Images were acquired by confocal laser scanning microscopy on a ZEISS LSM 880 mounted on an Axio Observer Z1 with a 63x/1.4 Plan‐Apochromat Oil DIC M27 Objective with an image size of 1024 × 1024 Pixels (51.90 μm × 51.90 μm) and12‐Bit depth. The bin value for the camera was set to 1 × 1. The optical section spacing between each z‐stack was approximately 0.15 μm. We used a 2.6 Scan Zoom. We captured three channels sequentially with the following parameters: γH2AX (excitation: 633 nm, emission: 668 nm, detection: 642–695 nm), Telomeres (excitation: 561 nm, emission: 617 nm, detection: 602–633 nm) and NucBlue (excitation: 405 nm, emission: 445 nm, detection: 410–479). IMARIS software 9.7 (Oxford Instruments) was used for image processing and TAF identification (Figure S4). Background subtraction, baseline subtraction and deconvolution were carried out for the telomere and the γH2AX channels individually. The nuclear staining channel was only deconvolved. In IMARIS, we created surfaces for telomeres, DNA damage foci (γH2AX staining) and nuclei. Then we created the overlap surface (where voxels inside each surface overlap with each other). We set our overlapped volume ratio to at least 1% overlap between γH2AX and telomere surfaces. Overlap of 1% or more between a telomere and a γH2AX surface was counted as a TAF. The overlap surface was labeled in yellow and corresponds to an individual TAF. NucBlue stain was used to delineate a single nucleus as a region of interest within which TAFs were counted. Very large surface luminal umbrella cells with very high γH2AX were identified as having 2+ TAFs by the IMARIS analysis and deemed to represent the TAF positive senescent cell population in the bladder. These cells were counted in lower magnification images acquired at 20X with an Axio Imager Z1 widefield fluorescent microscope (Cy5 Filter Excitation wavelength: 650 nm, 625‐655 nm, filter emission wavelength: 673 nm, 665–715; DAPI Filter Excitation wavelength: 353 nm, filter emission wavelength: 465 nm., Figure S5) and data presented as a percentage of these TAF positive cells relative to total urothelial cells in 3 different sections from 3 different bladders per age group. For the 3‐age group analysis, we used an ordinary one‐way ANOVA, with Tukey test for multiple comparisons. For comparing Vehicle and D + Q‐treatment we used an unpaired, two‐tailed *t* test (*p* < 0.05 was considered significant). Statistics done in GraphPad Prism 9.0.

### 4.9 Immunofluorescence

Cyclin D1 staining was carried out on bladders (*n* = 2 per group) obtained from middle aged (10‐months) and old (26‐months) female mice, and young, middle aged and old male mice (male data in Figure S8). Mice were euthanized by CO_2_ asphyxiation, bladders immediately excised and processed for paraffin embedding and sectioning (6 μm) as described above for RNAscope. Sections were baked for 1 h at 55°C, dewaxed in Histoclear (National Diagnostics, Charlotte, NC), hydrated with a decreasing ethanol gradient, washed with water, and heat‐mediated antigen‐retrieval carried out in 0.1 M citrate buffer, pH 6.0. After cooling and washing with water and PBS, sections were permeabilized and blocked for 1 h at RT in 0.3% Triton X100, 5% Normal goat serum in 1X PBS. Primary antibody (1:400 anti‐Cyclin D1 (E3P5S) XP® Rabbit mAb (Cell Signaling Technology, Cat. No. 55506) diluted in 1% BSA, 0.3% Triton X‐100 in 1X PBS) was added and incubated overnight at 4°C in a humidified chamber. No primary antibody controls were used. After washing in PBST (PBS + 0.05% Tween20), secondary antibody (1:500 Goat anti‐Rabbit IgG (H + L) Highly Cross‐Adsorbed Secondary Antibody, Alexa Fluor™ Plus 647 (Invitrogen, Cat. No. A32733) diluted in 1% BSA, 0.3% Triton X‐100 in 1X PBS) was applied and incubated for 1 h at RT. After PBST and PBS washes, slides were mounted with ProLong™ Diamond Antifade Mountant with DAPI (Invitrogen, Cat. No. P36962), coverslipped, dried overnight and imaged the next day using 4X and 20X objectives on an Olympus APEXVIEW APX100 microscope using settings for AF647 and DAPI fluorescence.

### 4.10 Voiding spot assays (VSAs)

Clean mouse cages were lined with Grade 1 Whatman filter paper (1001–929, GE Healthcare, UK) cut to fit the cage bottom. A single crumbled kimwipe was placed at the bottom of the cage for enrichment and to minimize paper chewing. Normal chow but no water was provided. A single mouse was placed in each cage and allowed to sit undisturbed for 4 h (between 11 am and 3 pm). VSA paper was imaged under UV using a ChemiDoc Biorad instrument and data analyzed using Void Whizzard, a FIJI (ImageJ) plugin (Wegner et al., 2018). Parameters compared included total number of voids, total void area, percentage of void area in cage corners, and percentage of large voids (>3 cm) of total voids. Statistics were as follows: Middle aged versus old, unpaired nonparametric Mann–Whitney *t* test, two‐tailed, NCD versus HFD, unpaired two‐tailed parametric *t* test, PBS versus Proliferating versus Senescent Cell transplantation and No treatment versus Water versus V versus D + Q, Ordinary one‐way ANOVA, with Dunnett's test for multiple comparisons (*p* < 0.05 was considered significant). Statistics done in GraphPad Prism 9.0.

### 4.11 Pressure/flow cystometry (CMG)

CMG was conducted as previously described (Smith et al., 2012; Smith & Kuchel, 2010) under urethane anesthesia, which uniquely preserves reflex voiding to continuous infusion, with minimal impact on cystometric parameters, yet permitting an unrestrained, still, anesthetized animal for study (Cannon & Damaser, 2001; Maggi & Conte, 1990; Smith et al., 2008). Mice were anesthetized with a 1 mg/mL intraperitoneal injection of urethane in PBS and allowed to sit in the dark for 30 min. Mice were further anesthetized with 1%–1.5% isoflurane for the duration of the catheterization procedure (15–25 min.). The bladder dome was intubated with a 10‐cm PE60 catheter with a flared tip and secured with a ligature. Animals were allowed 30–45 min to recover from the procedure and then placed prone with the urethral meatus overlying a 1.5‐cm hole, under which a tissue‐filled 5‐mL cup on a Grass FT03 force transducer was arranged to collect voided urine before droplet formation on the urethral meatus, 1 gm = 1 mL. An in‐line pressure transducer between a syringe pump and the bladder catheter recorded intravesical pressure. Infusion system was purged of air. Pressure and volume data were acquired during constant bladder infusion at 1.5 mL/h. Three sequential cycles following the establishment of a regular fill/void pattern were selected for analysis. Physiologic data were sampled at 30 Hz. LabChart8 (ADInstruments, Colorado Springs, CO) and Microsoft Excel were used for acquisition and analysis. Variables measured for each mouse were: Pbase (baseline pressure), Pthresh (threshold pressure at which the bladder switches from storages to voiding), P_max_ (maximum void‐associated pressure, occurs at the onset of void flow and relates detrusor voiding pressurization to urethral voiding function, previously associated with voiding spot size); IVI (intervoid interval, seconds between flow initiation of sequential voids), volume voided, mean flow rate, Pend (pressure at which the system switches from voiding to storage status and sphincter closes). Mean values of all CMG variables were calculated for each mouse from 3 consecutive voids. Mice were excluded from analysis when they didn't cycle, if their catheter was leaking and their belly was wet, if they died after anesthesia, if the bladder nick was too large so more than 1/4 of the bladder tissue was over the ligature. Statistics were as follows: Middle aged versus old, unpaired parametric two‐tailed *t* test with Welch's correction, NCD versus HFD and HFD versus HFD + D + Q, unpaired parametric two‐tailed *T* test, PBS versus Proliferating versus Senescent Cell transplantation and No treatment versus Water versus V versus D + Q, Ordinary one‐way ANOVA, with Dunnett's test for multiple comparisons (*p* < 0.05 was considered significant). Statistics done in GraphPad Prism 9.0.

### 4.12 Senescent cell transplantation

Female mouse ear fibroblasts (Jurk et al., 2014) were cultured and induced to senesce using 10 Gray of radiation as previously described (Xu et al., 2018) and used for the experiment 3 weeks after irradiation. An aliquot of cells was not irradiated, frozen and recultured a few days before the transplantation to be used as a nonsenescent (proliferating) cell control. Eighty 17‐months old female mice were injected intraperitoneally with 0.5–0.8 × 10^6^ proliferating cells, senescent cells or PBS (cells were resuspended in PBS, IP injection volume = 100 μL). Mice were ear notched and randomly assigned to treatment group and each cage contained mice treated with all three different conditions. Two months after cell transplantation, mice underwent bladder functional assays (VSAs on all mice and CMG (*n* = 10/group) as described earlier). Statistics: Ordinary one‐way ANOVA, with Dunnett's test for multiple comparisons, compared to PBS (*p* < 0.05 was considered significant). Statistics done in GraphPad Prism 9.0.

### 4.13 High‐fat diet

Six months old female mice were either fed a normal chow diet (NCD) or a high‐fat diet (HFD, 60% [by calories]) fat diet (D12492, irradiated; Research Diets, New Brunswick, NJ) for 3 months (20 mice total, *n* = 10 per group) and then underwent bladder functional assays (VSAs and CMG). A separate cohort of 20 female HFD fed mice was divided into two groups, one receiving vehicle and the other D + Q for a month, while staying on the HFD. V/D + Q (concentrations and content described earlier) was administered by oral gavage for three consecutive days every 2 weeks for 1 month. Mice then underwent bladder functional assays to test whether D + Q reversed the effect of HFD on bladder function.

## AUTHOR CONTRIBUTIONS

IAN conceived the study, designed and performed experiments, analyzed data and wrote the manuscript. MA, DB and LW performed experiments, analyzed data. LG, C‐LK analyzed data. MOF helped with interpretation of results. MX, PPS, and GAK helped with experimental design and interpretation of results. All authors reviewed the manuscript.

## CONFLICT OF INTEREST STATEMENT

M.X. has a financial interest related to this research: patents on senolytic drugs (including PCT/US2016/041646, filed at the US Patent Office) are held by Mayo Clinic. Other authors declare no conflict of interest.

## Supporting information


Appendix S1.

Appendix S2.

## Data Availability

All data is available by request to Iman Al‐Naggar (alnaggar@uchc.edu). RNA sequencing data has been deposited to the GEO at NCBI (Series GSE247123). https://urldefense.com/v3/__https://www.ncbi.nlm.nih.gov/geo/query/acc.cgi?acc=GSE247123__;!!Cn_UX_p3!jDUcDR‐Sg84pXUmv2MNrqVjRUx_Y47HyYkSOSE9Rt7ym‐ldLi3Ziio0qg5I3O53F3YwurNP0mdijBm6NJg$token:wpqzmogmvvibxyn.
