# Supplementary material for: Polyploid superficial uroepithelial bladder barrier cells express features of cellular senescence across the lifespan and are insensitive to senolytics
Source: Aging Cell. 2024 Dec 7;24(2):e14399. doi: 10.1111/acel.14399 (PMC11822673; doi:10.1111/acel.14399)
Supplement: Supplementary file 1 — Appendix S1. [file ACEL-24-e14399-s001.pdf]

SUP. FIG. 1

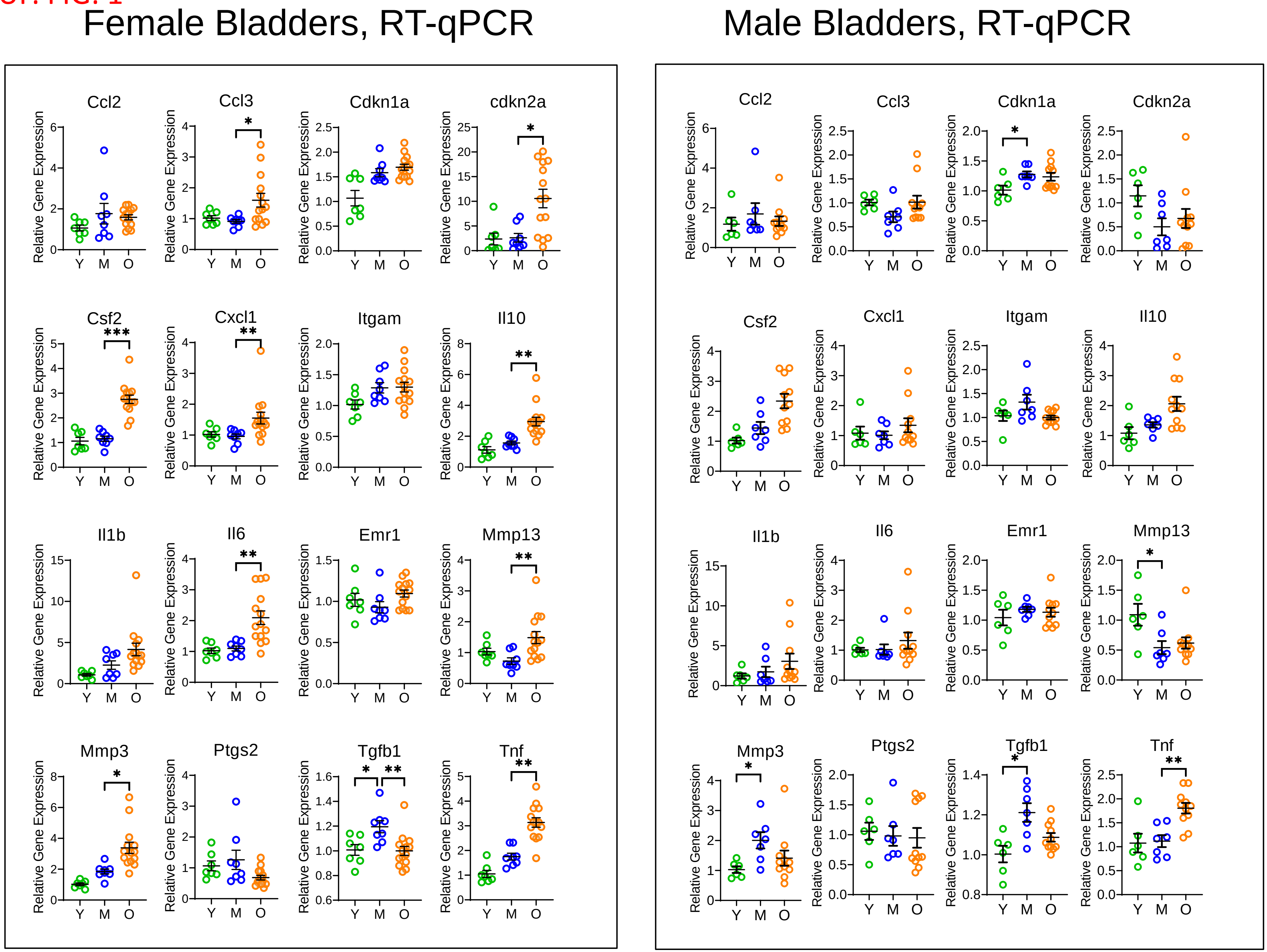
Female Bladders, RT-qPCR
Male Bladders, RT-qPCR
Ccl2
Ccl3
Cdkn1a
cdkn2a
Cdkn2a
Csf2
Cxcl1
Itgam
Il10
Il1b
Il6
Emr1
Mmp13
Mmp3
Ptgs2
Tgfb1
Tnf
Relative Gene Expression
Y
M
O

SUP. FIG. 2

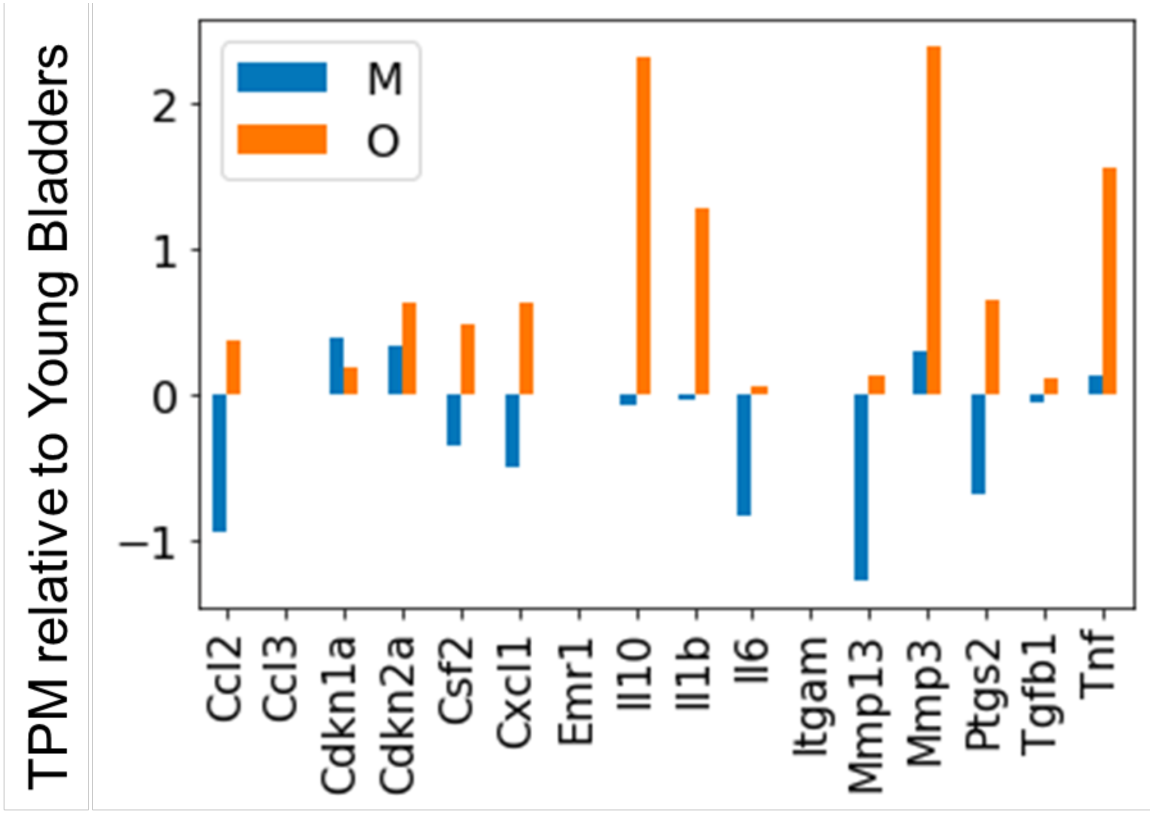

TPM relative to Young Bladders
M
O
2
1
0
−1
Ccl2
Ccl3
Cdkn1a
Cdkn2a
Csf2
Cxcl1
Emr1
Il10
Il1b
Il6
Itgam
Mmp13
Mmp3
Ptgs2
Tgfb1
Tnf

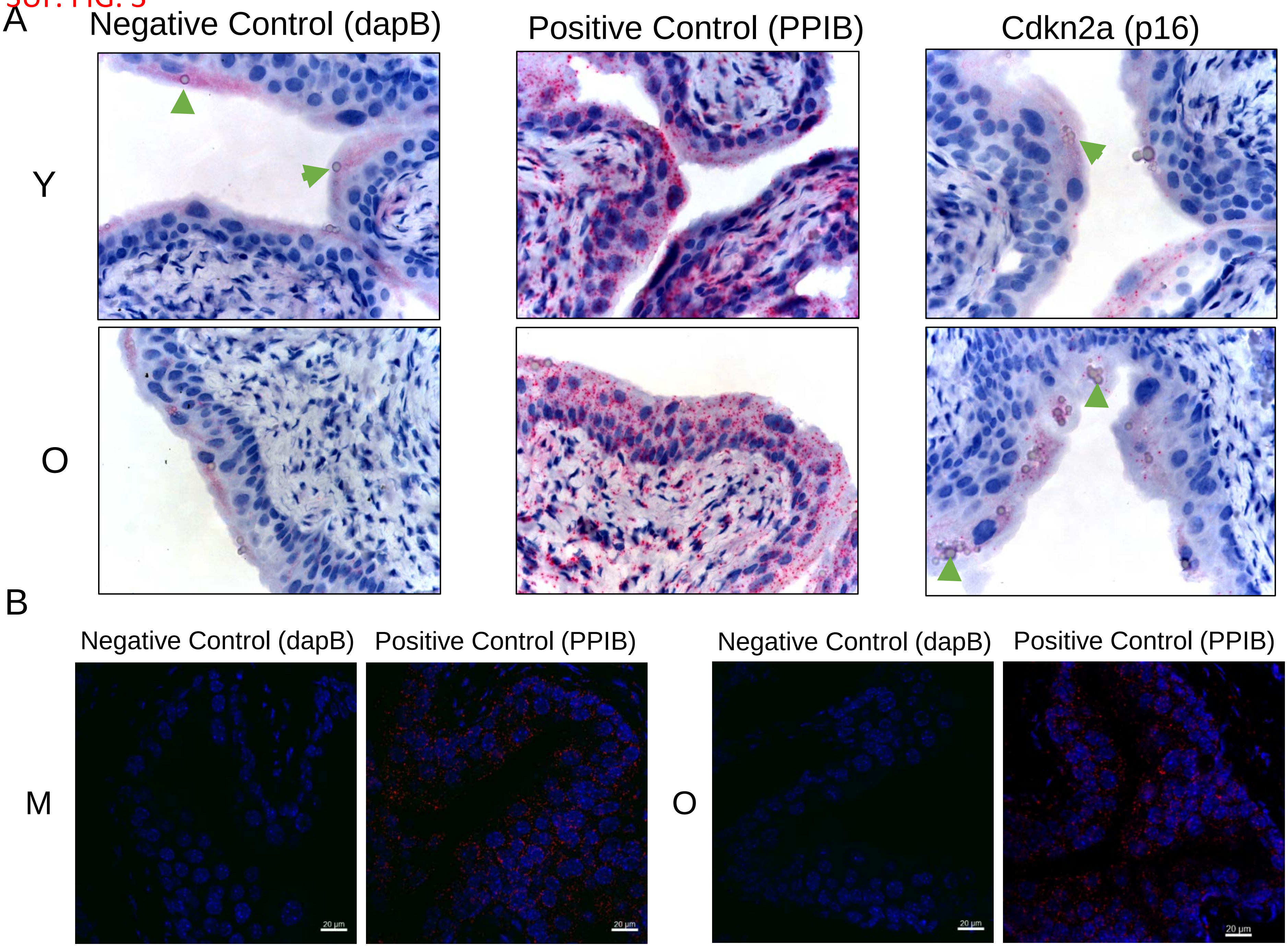
A
Negative Control (dapB)
Positive Control (PPIB)
Cdkn2a (p16)
Y
O
B
Negative Control (dapB)
Positive Control (PPIB)
Negative Control (dapB)
Positive Control (PPIB)
M
O
20 μm
20 μm
20 μm
20 μm

SUP. FIG. 4

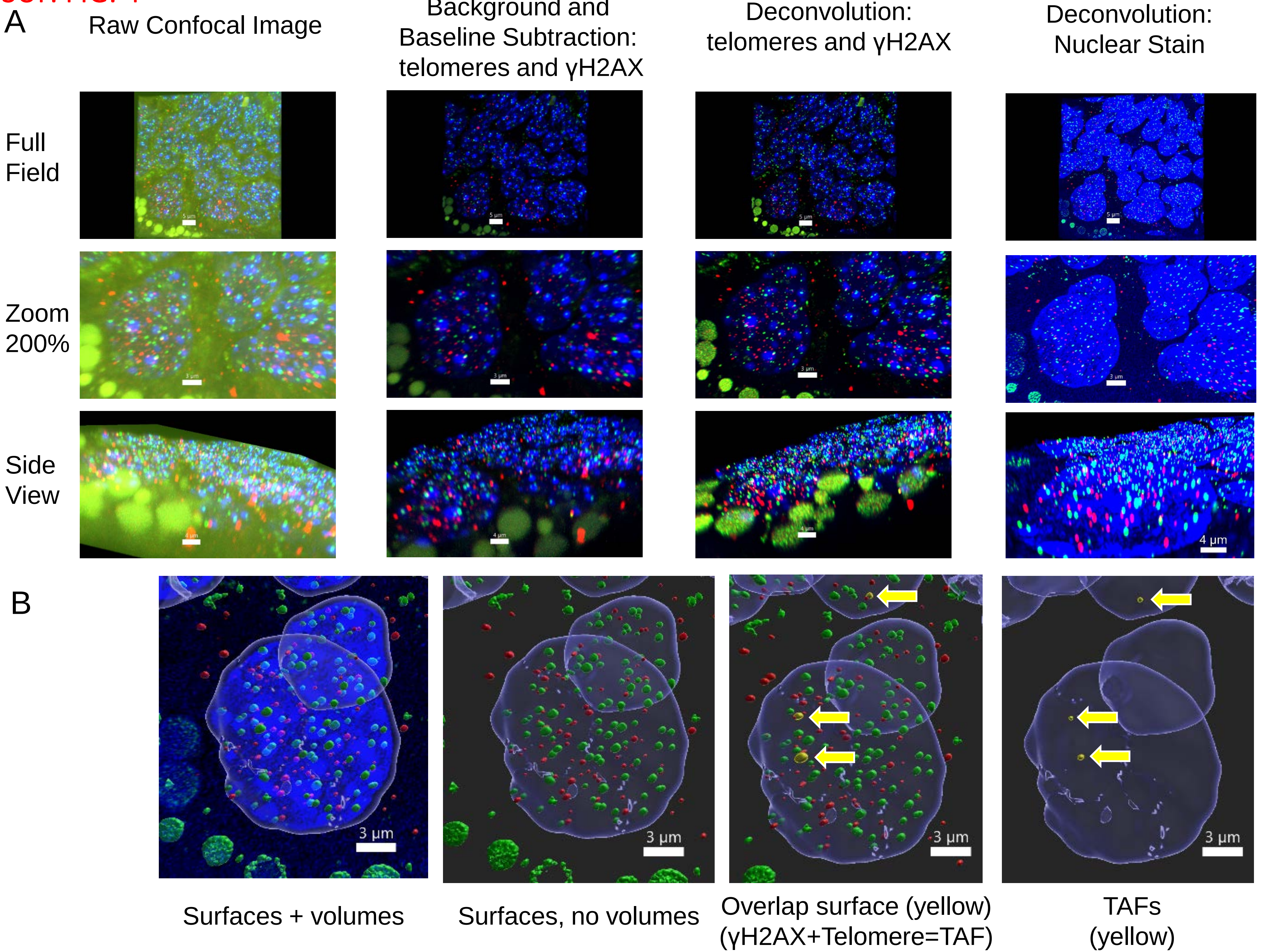
A
Raw Confocal Image
Background and Baseline Subtraction: telomeres and γH2AX
Deconvolution: telomeres and γH2AX
Deconvolution: Nuclear Stain
Full Field
Zoom 200%
Side View
4 µm
B
3 µm
3 µm
3 µm
3 µm
Surfaces + volumes
Surfaces, no volumes
Overlap surface (yellow) (γH2AX+Telomere=TAF)
TAFs (yellow)

SUP. FIG. 5

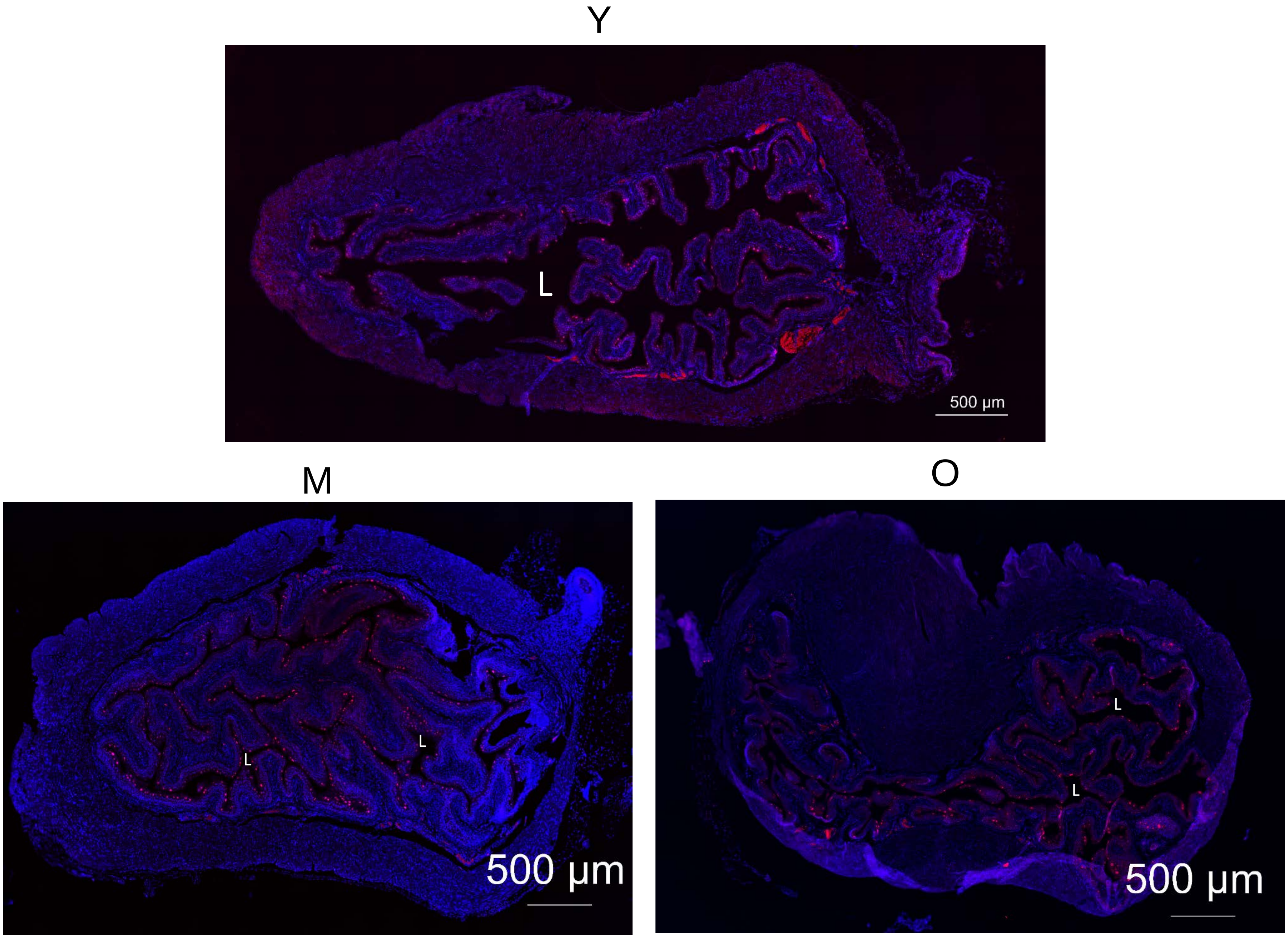

SUP. FIG. 6

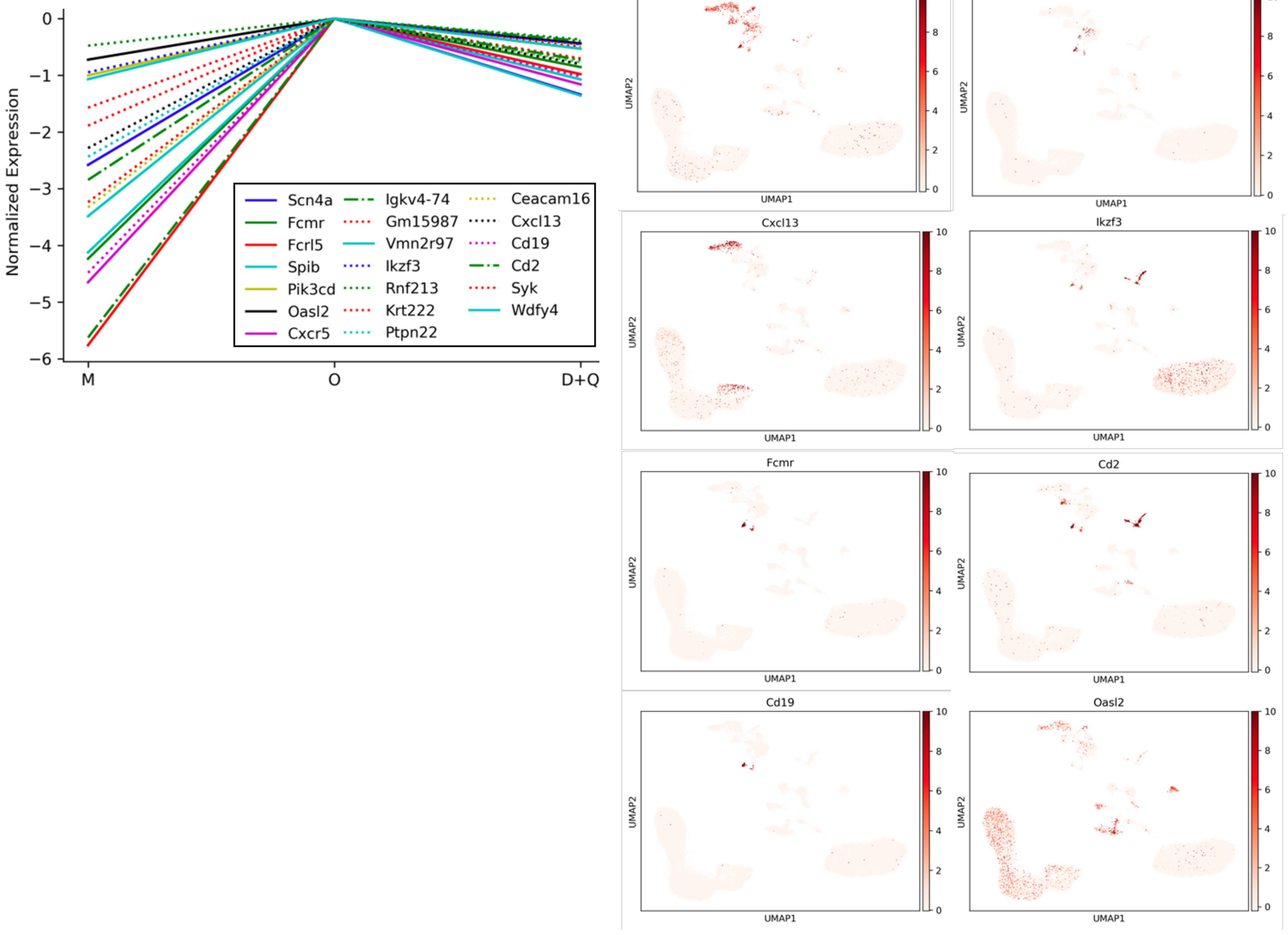
Normalized Expression
0
−1
−2
−3
−4
−5
−6
M
O
D+Q
Scn4a
Fcmr
Fcrl5
Spib
Pik3cd
Oasl2
Cxcr5
Igkv4-74
Gm15987
Vmn2r97
Ikzf3
Rnf213
Krt222
Ptpn22
Ceacam16
Cxcl13
Cd19
Cd2
Syk
Wdfy4
Syk
Spib
Cxcl13
Ikzf3
Fcmr
Cd2
Cd19
Oasl2
UMAP1
UMAP2
0
2
4
6
8
10

SUP. FIG. 7

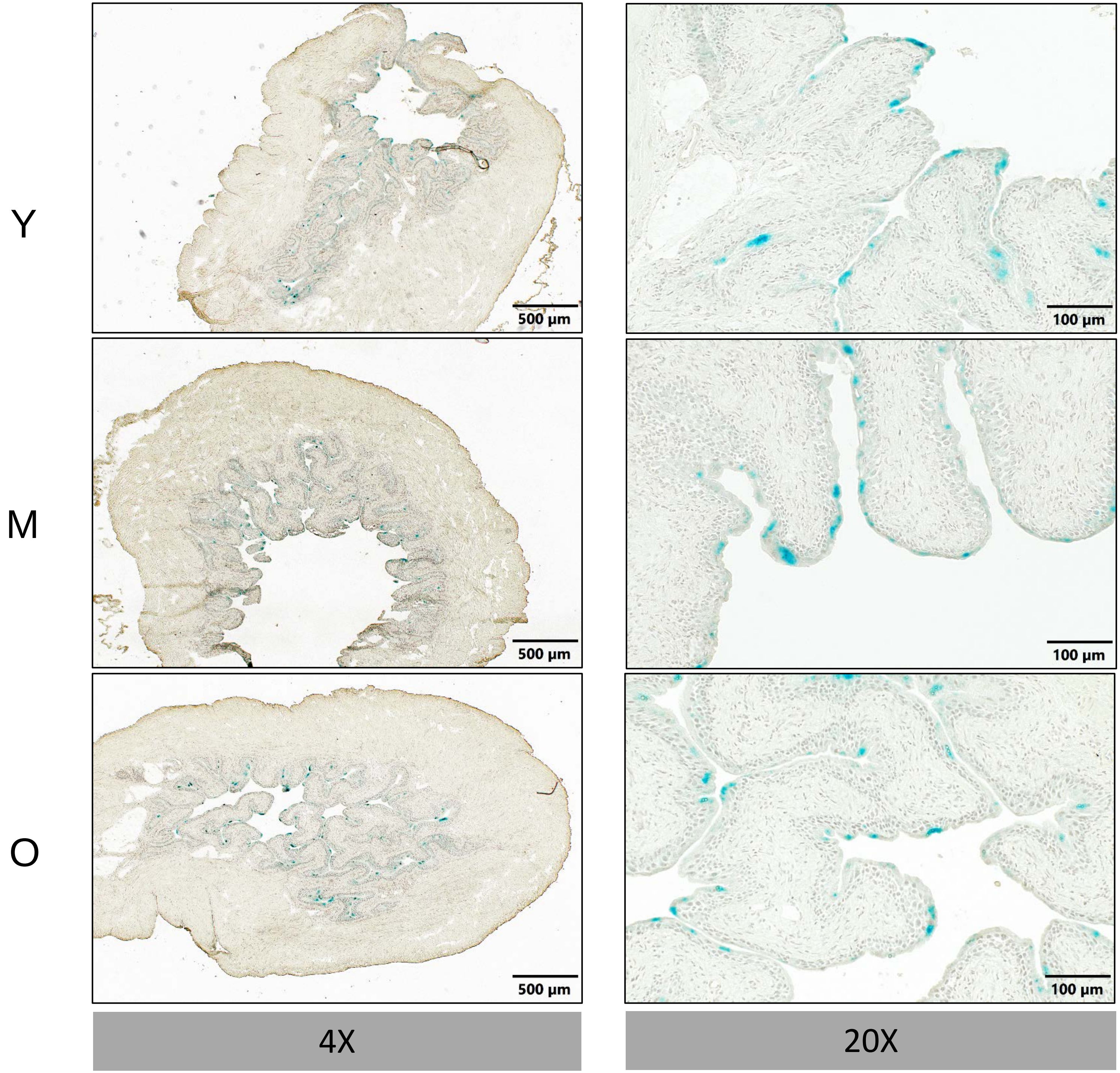

SUP. FIG. 8

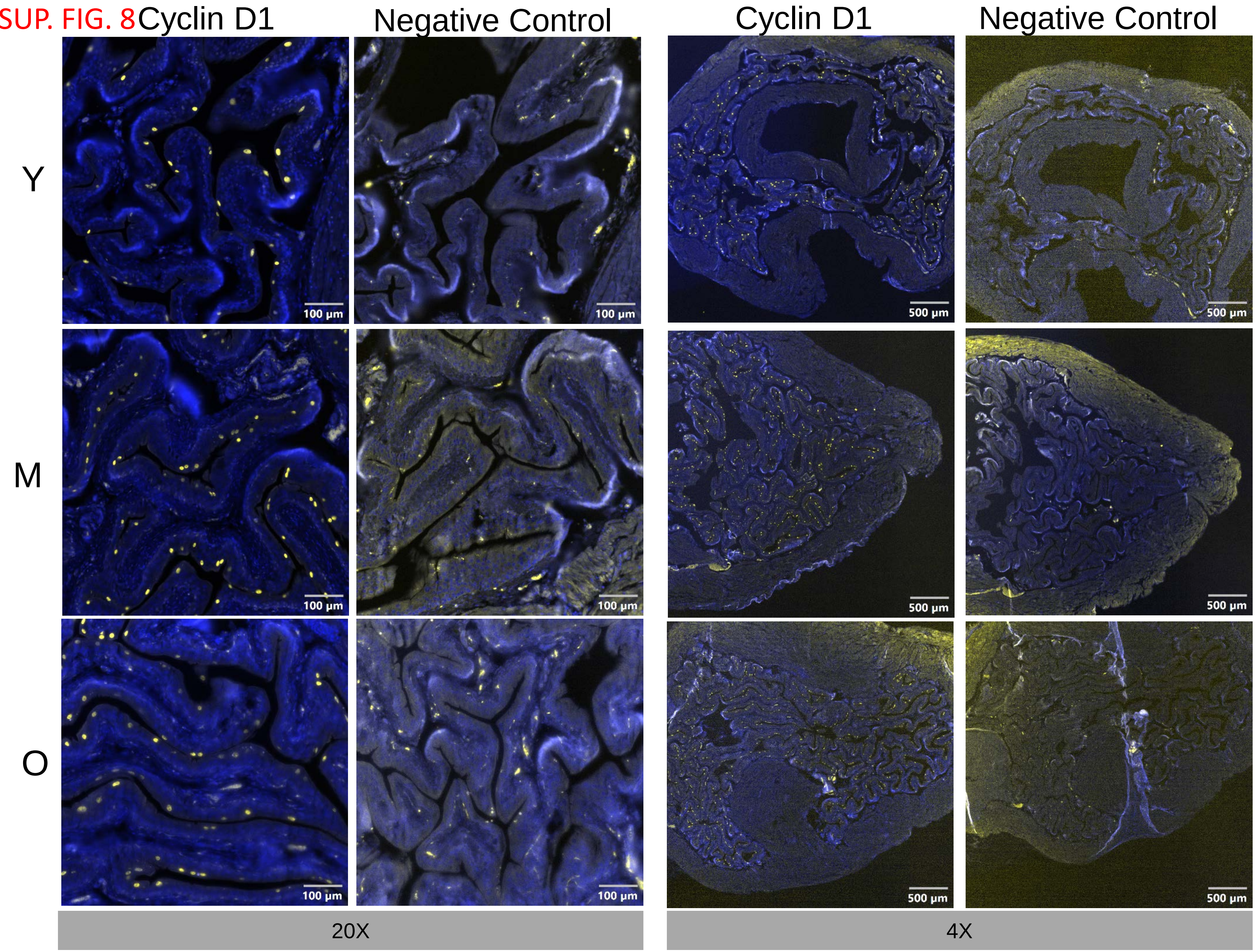
Cyclin D1
Negative Control
Cyclin D1
Negative Control
Y
M
O
100 µm
500 µm
20X
4X
