# Supplementary material for: Polyploid superficial uroepithelial bladder barrier cells express features of cellular senescence across the lifespan and are insensitive to senolytics
Source: Aging Cell. 2024 Dec 7;24(2):e14399. doi: 10.1111/acel.14399 (PMC11822673; doi:10.1111/acel.14399)
Supplement: Supplementary file 2 — Appendix S2. [file ACEL-24-e14399-s002.docx]

| **Gene Name** | **Gene Symbol** | **Ensembl Gene ID** | **Detected Coding Transcript(s)** | **Unique Assay ID** | **Role in Assay** |
| --- | --- | --- | --- | --- | --- |
| beta-2 microglobulin | B2m | ENSMUSG00000060802 | ENSMUST00000102476 | qMmuCID0040553 | Reference gene |
| ribosomal protein S18 | Rps18 | ENSMUSG00000008668 | ENSMUST00000080443, ENSMUST00000074072, ENSMUST00000008812, ENSMUST00000173871 | qMmuCED0045430 | Reference gene |
| tyrosine 3-monooxygenase/tryptophan 5-monooxygenase activation protein, zeta polypeptide | Ywhaz | ENSMUSG00000022285 | ENSMUST00000022894, ENSMUST00000110361, ENSMUST00000110362, ENSMUST00000126184, ENSMUST00000151635, ENSMUST00000110359 | qMmuCED0027504 | Reference gene |
| EGF-like module containing, mucin-like, hormone receptor-like sequence 1 | Emr1 | ENSMUSG00000004730 | ENSMUST00000086763, ENSMUST00000004850 | qMmuCID0017591 | Macrophage marker |
| integrin alpha M | Itgam | ENSMUSG00000030786 | ENSMUST00000064821, ENSMUST00000120355, ENSMUST00000098015, ENSMUST00000126475, ENSMUST00000106242 | qMmuCID0005971 | Leukocyte marker |
| chemokine (C-C motif) ligand 3 | Ccl3 | ENSMUSG00000000982 | ENSMUST00000001008 | qMmuCED0044190 | Senescence/SASP |
| prostaglandin-endoperoxide synthase 2 | Ptgs2 | ENSMUSG00000032487 | ENSMUST00000035065 | qMmuCED0047314 | Senescence/SASP |
| transforming growth factor, beta 1 | Tgfb1 | ENSMUSG00000002603 | ENSMUST00000002678 | qMmuCED0044726 | Senescence/SASP |
| tumor necrosis factor | Tnf | ENSMUSG00000024401 | ENSMUST00000025263, ENSMUST00000167924 | qMmuCED0004141 | Senescence/SASP |
| chemokine (C-C motif) ligand 2 | Ccl2 | ENSMUSG00000035385 | ENSMUST00000000193 | qMmuCED0003785 | Senescence/SASP |
| cyclin-dependent kinase inhibitor 1A | Cdkn1a | ENSMUSG00000023067 | ENSMUST00000119901, ENSMUST00000023829, ENSMUST00000122348 | qMmuCED0046265 | Senescence/SASP |
| cyclin-dependent kinase inhibitor 2A | Cdkn2a | ENSMUSG00000044303 | ENSMUST00000060501, ENSMUST00000107131, ENSMUST00000030237 | qMmuCED0038108 | Senescence/SASP |
| colony stimulating factor 2 (granulocyte-macrophage) | Csf2 | ENSMUSG00000018916 | ENSMUST00000019060 | qMmuCED0025728 | Senescence/SASP |
| chemokine (C-X-C motif) ligand 1 | Cxcl1 | ENSMUSG00000029380 | ENSMUST00000031327 | qMmuCED0047655 | Senescence/SASP |
| interleukin 1 beta | Il1b | ENSMUSG00000027398 | ENSMUST00000028881 | qMmuCID0005641 | Senescence/SASP |
| interleukin 6 | Il6 | ENSMUSG00000025746 | ENSMUST00000026845 | qMmuCID0005613 | Senescence/SASP |
| interleukin 10 | Il10 | ENSMUSG00000016529 | ENSMUST00000016673 | qMmuCID0015452 | Senescence/SASP |
| matrix metallopeptidase 3 | Mmp3 | ENSMUSG00000043613 | ENSMUST00000034497 | qMmuCID0018631 | Senescence/SASP |
| matrix metallopeptidase 13 | Mmp13 | ENSMUSG00000050578 | ENSMUST00000015394 | qMmuCID0025884 | Senescence/SASP |

**Supplementary Table 1:** Details of predesigned Biorad PrimePCR™ SYBR® Green Assays used for RT-qPCR and their targets.

|  | Highest Expression in Middle Age Compared to Old | Highest Expression in Old compared to Middle Age |
| --- | --- | --- |
| 1 | Hspa8 | Ighm |
| 2 | Actg1 | Igkc |
| 3 | Plin2 | Rpl35 |
| 4 | Ivl | Rpl36a |
| 5 | Egr1 | Rps4x |
| 6 | Hspa1b | Fau |
| 7 | Ly6d | Rpl30 |
| 8 | Fos | Rpl14 |
| 9 | Mal | Rpl36 |
| 10 | Hspb1 | Rpl38 |
| 11 | Dnajb1 | Rpl10 |
| 12 | Txnip | Thbs2 |
| 13 | Hspa1a | Rpl12 |
| 14 | Krt15 | Rpl13 |
| 15 | Btg2 | Rpl21 |
| 16 | Cebpb | Rpl11 |
| 17 | Krt19 | Sec61g |
| 18 | Dnaja1 | Rplp2 |
| 19 | Gsto1 | Rps12 |
| 20 | Elf3 | Rpl23 |

**Supplementary Table 2:** Differential gene expression across ages in luminal urothelial cells was performed by isolating luminal cells identified in *Baker et al.^28^* and performing a Wilcoxon rank sum test between middle aged and old bladders (n=4). Top 20 most highly expressed genes in luminal Visium spots from middle aged compared to old and old compared to middle aged female mouse bladders are listed in the table in order from highest to lowest differential expression. Visium spatial transcriptomics data was used from GSE180128^28^.
